# CoREST in pieces: Dismantling the CoREST complex for cancer therapy and beyond

**DOI:** 10.1126/sciadv.ads6556

**Published:** 2025-06-06

**Authors:** Houssam Ismail, Jalila Chagraoui, Guy Sauvageau

**Affiliations:** ^1^ Institute for Research in Immunology and Cancer (IRIC), Université de Montréal, Montréal, Québec, Canada.; ^2^ Department of Medicine, Université de Montréal, Montréal, Québec, Canada.; ^3^ Division of Hematology, Maisonneuve-Rosemont Hospital, Montréal, Québec, Canada.

## Abstract

Several landmark studies over the past decade have uncovered a critical role of the CRL3^KBTBD4^ ubiquitin ligase complex in regulating stability of corepressor of repressor element 1 silencing transcription factor (CoREST) complex proteins and normal hematopoietic stem cell self-renewal. There is now mounting evidence that the CoREST complex plays oncogenic roles, although the contributions of its catalytic versus noncatalytic functions remain unclear. Here, we summarize and discuss mechanisms whereby the CoREST complex coopts tissue-specific transcription factors to elicit pathogenic activity in cancer and neurodegenerative disease. We also identify tumor types with selective dependencies on the scaffolding properties of the CoREST complex. We argue that these tumor types may benefit from a KBTBD4-activating/CoREST complex degrader therapy, which could also enhance antitumor immunity and sensitize resistant tumors to immunotherapy. Overall, understanding how the CoREST complex operates abnormally and differences between its targeting through catalytic inhibitors or protein degraders will help discern all possible applications for targeting therapies now in clinical development.

## Teaser

CoREST complex proteins including LSD1 and CoREST1 play pathogenic roles in cancer and neurodegenerative diseases.

## INTRODUCTION

**Fig. 1.**
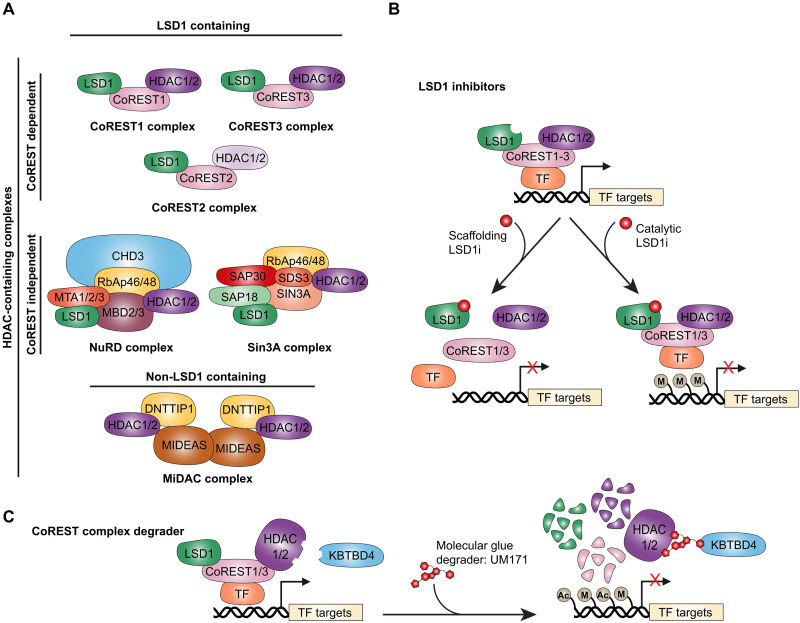
Therapeutic strategies targeting LSD1-containing HDAC complexes through LSD1 inhibitors or the CoREST complex degrader UM171. (**A**) The four major canonical HDAC1/2 multiprotein complexes can be grouped into those that contain LSD1 (CoREST, Sin3A, and NuRD) and those that do not (MiDAC). The CoREST complex consists of LSD1, CoREST1-3, and HDAC1 or HDAC2 and plays essential roles in neurogenesis, hematopoiesis, and cancer. The NuRD complex, composed of CHD3, MTA1-3, HDAC1/2, RbAp46/48, and MBD3, regulates hematopoiesis and maintains genomic stability (*22*). The Sin3A complex, which includes LSD1, HDAC1/2, SIN3A, SAP18/30, RbAp46/48, and SDS3, contributes to cell cycle control, embryogenesis, and tissue development (*20*). The MiDAC complex, which lacks LSD1, is composed of HDAC1/2 and ELM-SANT domain proteins such as DNTTIP1 and MIDEAS and is enriched in mitotic cells, where it regulates chromosome alignment and progression through mitosis (*24*, *25*). (**B**) LSD1 inhibitors are broadly classified into three mechanistic types. Catalytic inhibitors target LSD1’s FAD-binding site to block demethylase activity. Scaffolding inhibitors disrupt protein-protein interactions between LSD1 and key cofactors [CoREST or transcription factors (TF)], impairing complex assembly and chromatin recruitment. (**C**) UM171 represents a distinct therapeutic approach as a molecular glue degrader. It binds HDAC1/2 and the E3 ligase adapter protein KBTBD4, thereby activating KBTBD4 and promoting degradation of HDAC1/2-containing CoREST complexes (*27*–*30*). This leads to widespread increases in histone methylation and acetylation, resulting in altered transcriptional programs.

Therapeutic targeting of the epigenome has gained considerable momentum over the past decade given mounting evidence of frequent mutations and pathogenic implications of many chromatin-modifying enzymes. These enzymes work in concert with transcription factors and can write, read, or erase chromatin modifications, thereby influencing gene expression and controlling cellular function. Mammals express 18 histone deacetylase enzymes (HDACs), which are divided into four classes based on their homology to yeast HDACs as well as their subcellular localization (*1*). Closely related HDAC1 and HDAC2, both class I HDACs, are responsible for 60% of total cellular HDAC activity based on mouse knockout studies (*2*). Four main multiprotein canonical HDAC1/2 complexes have been discovered to date: (i) corepressor of repressor element 1 silencing transcription factor (CoREST), (ii) nucleosome remodeling and deacetylase (NuRD) complex, (iii) Sin3A, and (iv) mitotic deacetylase (MiDAC) complex (Fig. 1A).

Repressor element 1 silencing transcription factor (REST) was initially discovered in 1995 as a transcriptional repressor that binds neuron-restrictive silencer elements to silence neuronal gene expression in nonneuronal cells (*3*, *4*). This discovery identified REST as a master negative regulator of neurogenesis, with broad expression in nonneuronal tissues and higher levels in neuronal progenitors than in differentiated neurons (*4*). REST is a C2H2-type zinc finger domain protein that represses neuronal genes by recruiting chromatin-modifying complexes, including the CoREST complex (*5*–*7*). The CoREST complex consists of three core components: lysine demethylase 1 (LSD1; encoded by *KDM1A*), REST corepressor 1, 2, or 3 (CoREST1-3 encoded by *RCOR1-3*), and HDAC1. Furthermore, HDAC1 in the CoREST complex can form homodimers or heterodimers with HDAC2 (*8*). Additional subunits present in this complex include PHF21A, HMG20A/B, ZNF217, GSE1, and RREB1 (*9*).

CoREST proteins are not merely passive REST cofactors but can also have REST-independent roles during neurogenesis (*10*, *11*). CoREST1 is essential for maintaining the repression of pluripotency in neural stem cells (NSCs), and its depletion leads to premature differentiation (*12*). Furthermore, genome-wide studies have shown that CoREST1 binds to both REST-dependent and REST-independent targets in NSCs, suggesting unique regulatory functions (*12*). CoREST1, CoREST2, and CoREST3 are structurally similar proteins that all interact with LSD1 (*13*). However, while both CoREST1 and CoREST3 can strongly recruit HDAC1/2 to assemble a functional CoREST repressor complex, CoREST2 associates with the complex with lower affinity (*13*).

LSD1, the first discovered lysine demethylase, is a flavin-dependent monoamine oxidase that demethylates mono- and dimethylated H3K4 or H3K9 (H3K4me1/2 or H3K9me1/2), depending on its associated transcription factors or cofactors, thereby functioning as either a transcriptional repressor or activator (*14*). CoREST1 enhances LSD1 activity by tethering it to nucleosomes via its SANT2 domain, facilitating efficient demethylation of H3K4me1/2 (*15*). CoREST1 also promotes LSD1 protein stability (*15*). In addition, LSD1 and HDAC1 mutually enhance each other’s enzymatic functions within the CoREST complex (*16*), where CoREST1, through its flexible bilobed structure, binds both enzymes and coordinates their activities (*17*). In contrast, CoREST3 has been shown to suppress LSD1 demethylase activity, suggesting that the specific composition of CoREST complexes can influence their transcriptional output (*18*).

While LSD1 also associates with CoREST-independent complexes such as Sin3A and NuRD (Fig. 1A), genome-wide chromatin immunoprecipitation sequencing data reveal that ~86% of LSD1 binding sites overlap with CoREST1 and HDAC1, indicating that most of the LSD1 are found in CoREST complexes (*19*). Whether this high degree of overlap is conserved across cell types is still unknown. The roles of LSD1 in Sin3A and NuRD complexes remain underexplored. The Sin3A complex, which contains HDAC1/2, regulates processes such as the cell cycle, embryogenesis, and tissue development (*20*). In breast cancer cells, LSD1 partners with Sin3A to regulate growth, epithelial-to-mesenchymal transition (EMT), and chemosensitivity (*21*). On the other hand, the NuRD complex—which includes CHD3, MTA1-3, HDAC1/2, RbAp46/48, and MBD3—controls hematopoiesis and genomic stability (*22*). LSD1’s association with NuRD modulates transforming growth factor–β1 (TGF-β1) signaling to suppress invasion and metastasis in triple-negative breast cancer (*23*). Because most studies rely on LSD1 knockdown or inhibition rather than dissecting complex-specific functions, the contribution of CoREST-bound LSD1 to these mechanisms remains unclear. Additional genome-wide studies are needed to specifically examine the cistromes of each of these LSD1-containing complexes and delineate unique versus overlapping genomic binding and transcriptional outputs.

The MiDAC complex has been described more recently and is composed of HDAC1/2 and ELM-SANT domain–containing proteins, such as DNTTIP1 and MIDEAS (Fig. 1A). It is notably enriched in cells arrested in mitosis, where it plays a role in chromosome alignment and mitotic progression (*24*, *25*). Unlike the CoREST or NuRD complexes, MiDAC is not known to associate with LSD1, suggesting that it operates through distinct mechanisms of chromatin regulation.

Members of the CoREST complex play pathogenic roles in both solid and blood cancers as well as a number of other diseases such as brain and metabolic disease. Ongoing efforts have largely focused on individual targeting of LSD1 or HDAC proteins via small-molecule inhibitors. A number of LSD1 inhibitors are now being examined in clinical trials for treatment of essential thrombocythemia and a number of solid and blood cancers. LSD1 inhibitors can be broadly categorized into three types based on their mechanism of action (Fig. 1, B and C). Catalytic inhibitors block LSD1’s demethylase activity by covalently or noncovalently targeting its flavin adenine dinucleotide (FAD)–binding site. Scaffolding inhibitors function as protein-protein interaction disruptors, interfering with LSD1’s interactions with cofactors such as CoREST or GFI1/1B, thereby altering its recruitment to chromatin. A third recently described class, known as molecular glue degraders, induces the degradation of LSD1 protein, eliminating both its catalytic and scaffolding functions (Fig. 1C). In 2014, our group developed the UM171-optimized compound, a pyrimidoindole derivative, as a potent agonist of CD34^+^ hematopoietic stem cell (HSC) self-renewal ex vivo (*26*). UM171 is now being assessed in several clinical trials of transplantation of ex vivo–expanded HSCs in adult and pediatric patients with severe blood malignancies in Canada, Europe, and the US. Through genome-wide CRISPR-Cas9 knockout screens, we identified KBTBD4, a kelch-BTB domain adapter protein to the Cullin 3 ubiquitin ligase (CRL3), as the functional target of UM171 (*27*). We and others have recently shown that UM171, which acts as a KBTBD4-HDAC1/2 molecular glue, induces strong and rapid KBTBD4-dependent proteasomal degradation of all core members of the CoREST1/3 complex, thereby providing a potential therapy with a wide range of treatment indications (Fig. 1C) (*27*–*30*). Whereas UM171 remains the only known potent degrader of all CoREST1/3 complex proteins, two CoREST complex–selective HDAC inhibitors (SR-4370 and TNG260) have been described (*31*, *32*).

To date, no potent LSD1 inhibitors have been approved for clinical use. However, the antidepressants tranylcypromine (TCP) and phenelzine sulfate—originally developed as monoamine oxidase inhibitors—were later found to weakly inhibit LSD1. In addition, a number of HDAC inhibitors have been approved by the Food and Drug Administration for treatment of cutaneous (vorinostat and romidepsin) or peripheral (romidepsin and belinostat) T cell lymphoma and multiple myeloma (panobinostat). While small-molecule inhibitors ensure blockade of the demethylase/deacetylase activity of the CoREST complex, recent data discussed below suggest that this may not equate to its targeting via selective protein degradation. Indeed, LSD1 and HDAC enzymes also harbor demethylase- or deacetylase-independent activities.

In this review, we provide the most comprehensive synthesis to date of the pathogenic roles of the CoREST complex in human disease, based on an extensive survey of the literature. We highlight how CoREST complex proteins contribute to diverse tumor phenotypes, explore their dysregulated mechanisms of action across neurodegeneration and immunity, and examine emerging clinical data. Particular emphasis is placed on the distinction between chemical inhibition and targeted protein degradation and how these approaches may elicit divergent cellular responses. We also discuss recent insights into KBTBD4 as a master regulator of the CoREST complex, offering a mechanistic link between CoREST complex protein degradation and transcriptional reprogramming. Together, these findings underscore the importance of understanding the nuanced biology of the CoREST complex to fully unlock its therapeutic potential.

## CoREST TARGETING IN BLOOD CANCERS

**Fig. 2.**
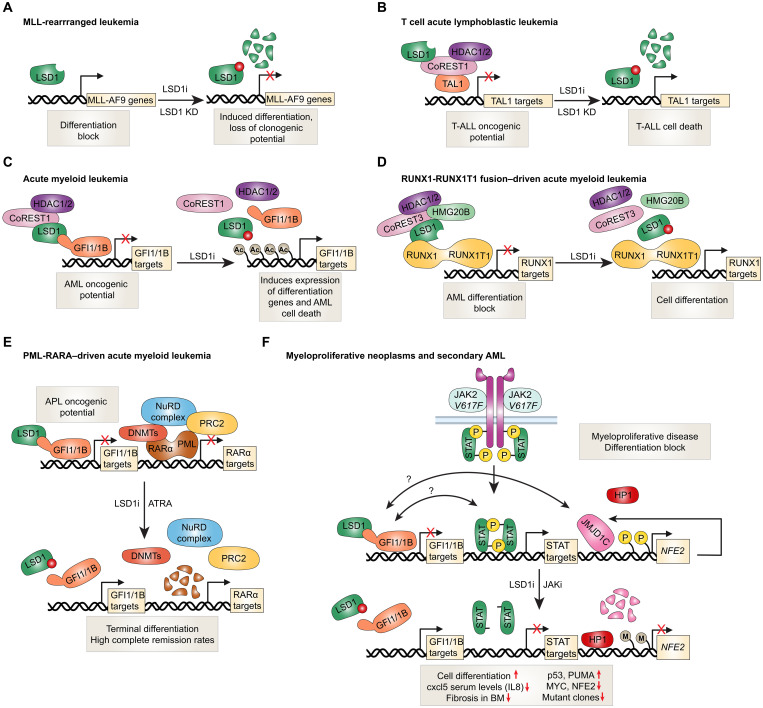
Mechanistic roles of LSD1 and the CoREST complex in hematologic malignancies. (**A**) In MLL-AF9–driven leukemia, LSD1 sustains leukemic stem cell identity by maintaining expression of MLL-AF9 target genes. LSD1 knockdown leads to accumulation of H3K4me2 at MLL-AF9–bound loci, paradoxically resulting in gene repression, induced differentiation, and loss of leukemic transcriptional programs (*37*). (**B**) In T-ALL, aberrantly overexpressed TAL1 recruits the CoREST complex to mediate transcriptional repression. LSD1 is required for TAL1 function, and its loss leads to derepression of TAL1 target genes (*40*, *41*). (**C**) In AML, the CoREST complex partners with transcriptional repressors GFI1 and GFI1B, reinforcing the differentiation block. LSD1 and CoREST1 co-occupy GFI1-bound chromatin in MLL-rearranged AML, and LSD1 inhibition disrupts this complex, increases histone acetylation, and promotes myeloid differentiation (*48*). (**D**) In RUNX1-RUNX1T1–driven AML, LSD1 inhibition results in eviction of LSD1, CoREST1, and HMG20B from chromatin, derepressing genes involved in differentiation (*55*). (**E**) In APL, the PML-RARα fusion protein recruits chromatin repressors including NuRD, PRC2, and DNMTs (*56*). LSD1 can also recruit GFI1/1B to repress differentiation genes. Pharmacologic doses of ATRA degrade the fusion protein, restoring differentiation and inducing remission. ATRA also synergizes with LSD1 inhibitors that disrupt GFI1/1B interactions to induce cell differentiation (*57*). (**F**) In MPNs, the LSD1 inhibitor bomedemstat promotes apoptosis and differentiation of *JAK2*^V617F^ -mutant cells (*62*–*64*). This occurs through disruption of GFI1/1B interactions and up-regulation of p53 and PUMA. *JAK2*^V617F^ signaling drives NFE2 overexpression via H3Y41 phosphorylation, which blocks binding of the repressor HP1 at the *NFE2* promoter (*65*). NFE2 also induces JMJD1C, reinforcing its own expression. JAK2 inhibition restores HP1 binding, suppressing both NFE2 and JMJD1C. Although direct cooperation between LSD1 and this axis remains unclear, bomedemstat reduces NFE2 levels, suggesting convergence with JAK-STAT signaling. BM, bone marrow; KD, knockdown; JAKi, JAK inhibitor.

The CoREST complex plays pathogenic roles in leukemia primarily through association with oncogenic chromatin remodeling or transcription factors. Perhaps one of the best known examples of diseases with prominent histone methylation aberrations is that of acute myeloid leukemia (AML) with translocations in the *MLL* gene that encodes for an H3K4 methyltransferase. MLL-rearranged leukemia shows poor prognosis. In addition, H3K4 methylation is critical for hematopoiesis (*33*). MLL increases H3K4 histone methylation at upstream regulatory regions of clustered homeobox (HOX) genes essential for hematopoietic differentiation (*33*). Indeed, we have previously reported that maintenance of optimal levels of HOX genes is crucial as their overexpression can perturb HSC proliferation and differentiation but also can be leukemogenic in mice (*34*–*36*). LSD1 has been reported to control the differentiation block in MLL leukemia by supporting expression of oncogenic MLL-AF9 target genes in leukemic stem cells (Fig. 2A) (*37*). LSD1 knockdown resulted in an increase in LSD1-regulated H3K4me2 marks at MLL-AF9–bound loci, which, unexpectedly, were accompanied with repression of MLL-AF9 target genes (*37*). This suggested that MLL-AF9 coopts LSD1 to maintain expression of its oncogenic repertoire. Consistent with this, targeting LSD1 through short hairpin RNA–mediated knockdown or through more potent analogs of TCP induced differentiation as well as loss of clonogenic potential of murine and human MLL leukemia cells both ex vivo and in vivo (*37*). Similar results have also been observed with other LSD1 inhibitors (*38*).

Aberrant overexpression of the TAL1 oncogenic transcription factor is found in ~50% of T cell acute lymphoblastic leukemias (T-ALL) and is associated with less favorable outcomes (*39*). TAL1’s oncogenic potential appears to arise in part through association with the CoREST complex. LSD1 is necessary for TAL1-mediated transcriptional repression, and knockdown of LSD1 results in derepression of TAL1 target genes in T-ALL (Fig. 2B) (*40*, *41*). The contribution of CoREST1 and HDAC1/2 to TAL1 activity remains unclear, although their association with TAL1 is reduced at the onset of culture-induced differentiation and is then restored at late stages. Transgenic mice overexpressing the shortest isoform of LSD1 in hematopoietic stem/progenitor cells (HSPC) developed T-ALL at much higher frequency upon exposure to γ-irradiation (*42*). It remains unclear whether overexpression of CoREST1 or HDAC1/2 in mice would result in a similar phenotype or even tumors of nonhematopoietic origin. Interestingly though, these murine T-ALL tumors, which maintain LSD1 overexpression, carried NOTCH1 activating mutations, which are an important feature of more than 50% of human T-ALL cases (*42*, *43*). TCP analogs with improved LSD1 inhibitory activities showed efficacy against T-ALL cells both ex vivo and in vivo (*44*). Together, elevated LSD1 levels can prime HSPCs for leukemogenesis, and whether there is any contribution of other CoREST complex proteins in this mechanism remains to be seen.

In the hematopoietic system, the CoREST complex partners with the transcriptional repressors GFI1 and GFI1B, which are critical for the endothelial-to-hemogenic transition and for the differentiation of erythroid and megakaryocytic lineages (*45*–*47*). In AML, the CoREST-GFI1/GFI1B complex has been well characterized as a key mediator of the differentiation block that sustains the leukemic state (Fig. 2C). In THP1 cells that harbor a t(9;11) *MLL* translocation, a notable 71% of GFI1 chromatin immunoprecipitation peaks overlapped with LSD1, and ~33% overlapped with CoREST1 binding (*48*). Importantly, almost all of the strongest GFI1 peaks overlapped with both LSD1 and CoREST1 peaks (*48*). GFI1/GFI1B bind LSD1 potentially in its catalytic site through their N-terminal SNAG domains (*45*, *49*). Treatment with OG86, an LSD1 inhibitor, reduces AML cell clonogenic potential through disrupting the interaction between GFI1 and the CoREST complex on chromatin (*48*). This consequently enhances activating histone acetylation marks presumably through release of HDAC1/2. This in turn induces expression of transcription factors necessary for expression of myeloid differentiation genes. Interestingly, while these results could be recapitulated with LSD1 knockdown, forced expression of the K661A LSD1 demethylase-dead mutant was able to restore AML cell clonogenic potential and reestablish the differentiation block. This differentiation block could also be seen with ectopic expression of a GFI1-LSD1 fusion protein in the presence of LSD1 inhibitors, which highlights the importance of the association between these two factors. CRISPR scanning screens to identify mutations in LSD1 that confer resistance to LSD1 inhibitors have revealed that LSD1 demethylase activity is not needed for AML cell survival (*50*). Instead, the antiproliferative properties of LSD1 inhibitors are mediated through disrupting the interaction between GFI1 and LSD1. These results demonstrate that the scaffolding—and not catalytic—properties of LSD1 are responsible for GFI1-mediated differentiation blocks uniformly seen in AML. However, another report has revealed that conditional knock-in of a catalytically inactive LSD1 mutant did lead to morphological signs of differentiation in murine Hoxa9/Meis1-driven AML cells (H9M) (*51*). Importantly though, this mutant did not impart any survival benefit in H9M cell transplantation experiments, whereas a full LSD1 knockout did (*51*). Thus, it may be that the disruption of both demethylase and scaffolding properties of LSD1 is necessary to confer therapeutic benefit in certain cellular contexts or AML models.

Aberrant recruitment of HDAC1/2 by oncogenic fusion proteins in AML is also a well-described pathogenic mechanism. For instance, *RUNX1-RUNX1T1* (AML1-ETO) and PML-RARα oncogenic fusion proteins recruit HDAC1/2 to repress RUNX1 and RARα target genes, respectively, which contributes to the differentiation block in AML (*52*). Whether HDAC1/2 specific to CoREST complexes are involved in this mechanism is unknown, although LSD1, HDAC1/2, CoREST3, and HMG20B have been shown to copurify with AML1-ETO fusion proteins (*53*). Furthermore, GFI1 is highly expressed in *RUNX1-RUNX1T1*–driven leukemic cell lines and is required for their survival (*54*). Indeed, *RUNX1-RUNX1T1*–driven leukemic cell lines were among the most sensitive to several LSD1 inhibitors or LSD1 knockdown in a panel of AML, chronic myelogenous leukemia, and T-ALL cell lines (*55*). Mechanistically, LSD1 inhibitors caused eviction of LSD1 together with CoREST1 and HMG20B from a fraction of LSD1 target genes involved in cell differentiation (Fig. 2D) (*55*).

In the case of PML-RARα–driven AML, also referred to as acute promyelocytic leukemia (APL), the PML-RARα fusion protein exerts its oncogenic activity by aberrantly recruiting NuRD, PRC2, and DNMT proteins, leading to transcriptional repression of genes critical for myeloid differentiation and contributing to the characteristic differentiation block of APL (Fig. 2E) (*56*). All-trans retinoic acid (ATRA) treatment at pharmacological doses causes degradation of the PML-RARα fusion protein, which results in terminal leukemic cell differentiation, and high complete remission rates in patients. While this form of leukemia is resistant to LSD1 inhibitors or LSD1 depletion, LSD1 inhibition sensitized APL cells to low physiological concentrations of ATRA, and cotreatment substantially enhanced survival in leukemic mice (*57*). Importantly, ATRA treatment is not effective in non-APL cells. However, LSD1 inhibitor or knockdown treatment can also potentiate ATRA-induced differentiation in non-APL cells (*58*). Cotreatment with ATRA and LSD1 inhibitors was associated with reduced engraftment of primary human AML in mice and was superior to treatment with either drug alone (*58*). Induction of cell differentiation in APL or non-APL cells was independent of LSD1’s catalytic activity and involved disrupting its interaction with GFI1 (*57*).

Overall, the individual contribution of other members of the CoREST complex in the oncogenic roles of GFI1 and other pathogenic factors in leukemia remains unclear. A combination of LSD1 and HDAC inhibitors was superior in extending survival of mice engrafted with human AML compared to either agent alone (*59*). Thus, on the basis of the above, it is plausible that simultaneously targeting all members of the CoREST complex—especially through degradation to abrogate scaffolding properties—could more profoundly disrupt the oncogenic programs governed by GFI1 and other pathogenic factors in leukemia.

Myeloproliferative neoplasms (MPNs) represent a group of clonal malignant hematopoietic disorders that include primary myelofibrosis, essential thrombocythemia, and polycythemia vera. All three types of MPNs can transform and progress to AML, which is associated with substantial mortality. MPNs are driven by activating *JAK2* and *CALR* mutations that result in hyperactive signal transducers and activators of transcription (STAT) signaling. Overexpression of nuclear factor erythroid 2 (NFE2), a key transcription factor involved in platelet production, or recurrent somatic mutations resulting in a hyperactive, truncated form of NFE2 have been directly linked to the MPN phenotype. These alterations are associated with thrombocytosis, leukocytosis, reticulin fibrosis, and expansion of malignant stem/progenitor compartments and can progress to spontaneous transformation to AML (*60*, *61*).

LSD1 has recently emerged as a compelling therapeutic target in MPNs. This interest is rooted in the LSD1’s dual role in regulating epigenetic plasticity and HSC fate. In contrast to Janus kinase (JAK) inhibitors, which primarily control symptom burden and splenomegaly, LSD1 targeting offers the potential for disease modification by blocking the malignant stem cell compartment and the underlying transcriptional programs driving disease persistence and/or progression. One of the most advanced agents in this class is bomedemstat (IMG-7289), an irreversible LSD1 inhibitor now under clinical evaluation. Preclinical models of MPN have demonstrated that bomedemstat not only reduces spleen size and normalizes blood counts but also lowers mutant allele burden and improves survival by promoting differentiation and apoptosis of *JAK2*^V617F^ cells (*62*). These effects are attributed to both LSD1 demethylase activity and disruption of GFI1/1B interactions, which result in up-regulation of p53 and PUMA and suppression of BCLXL (Fig. 2F) (*62*–*64*). *JAK2*^V617F^ signaling contributes to NFE2 overexpression by increasing H3Y41 phosphorylation at the *NFE2* gene promoter, which disrupts binding of the HP1 repressive protein (*65*). NFE2 also positively regulates expression of the histone demethylase JMJD1C, which further contributes to NFE2 expression (*65*). JAK2 inhibition inhibits H3Y41 phosphorylation and allows HP1 binding resulting in suppressed expression of NFE2 and JMJD1C (*65*). While it remains unknown whether LSD1 directly cooperates with JAK-STAT signaling in MPNs, evidence suggests potential points of convergence. Specifically, LSD1, STAT5, and HDAC3 have been shown to interact and co-occupy chromatin (*66*), and bomedemstat has been reported to suppress NFE2 expression (*62*)—although it is unclear whether this involves cross-talk with the JMJD1C-HP1 regulatory axis. Early clinical evidence in patients with myelofibrosis and essential thrombocythemia suggests that bomedemstat is well tolerated and capable of reducing the frequency of mutant clones (*63*) and may be effective in post-MPN AML transformation, as illustrated in a case study of prolonged remission following treatment (*67*).

Another promising agent is JBI-802, the first-in-class dual LSD1/HDAC6 inhibitor to enter clinical trials. JBI-802 combines the epigenetic modulatory effects of LSD1 inhibition with the epigenetic and cytoplasmic regulatory functions of HDAC6 targeting, aiming to maximize antileukemic activity. Preclinical models have demonstrated robust antiproliferative effects and enhanced synergy with immune checkpoint blockade in solid tumors and hematologic malignancies. JBI-802 is now being tested in phase 1/2 trials for patients with advanced solid tumors, MPNs, and myelodysplastic/MPN overlap syndromes (*68*).

Beyond monotherapy, LSD1 inhibitors are being explored in rational combinations. For example, coinhibition of LSD1 and BRD4 (BETi), or LSD1 and JAK2 (ruxolitinib), shows synergistic efficacy in AML and secondary AML post-MPN by disrupting enhancer regulation and overcoming nongenetic resistance mechanisms (*64*). These combinatorial approaches may be particularly relevant in high-risk or treatment-refractory myelofibrosis, where JAK inhibitors alone are insufficient to halt leukemic transformation.

## CoREST TARGETING IN BREAST CANCER

**Fig. 3.**
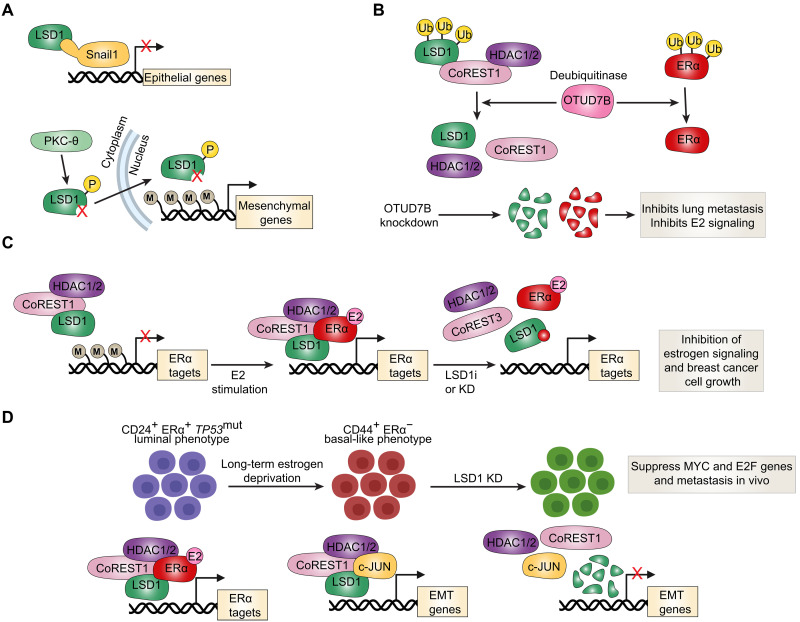
Roles of the CoREST complex in regulating EMT, hormone signaling, and endocrine therapy resistance in breast cancer. (**A**) In breast cancer, LSD1 and CoREST1 expression correlate with the EMT regulator Snail1, which recruits LSD1 via its SNAG domain to repress epithelial gene expression and promote migration. This interaction is stabilized by CoREST1 and can be disrupted by LSD1 inhibitors (*49*). Phosphorylation of LSD1 at serine 111 (S111) by PKC-θ enhances its nuclear localization and reduces its demethylase activity, leading to increased expression of EMT markers (*69*). Together, these mechanisms position LSD1 as a central regulator of EMT and CSC programs. (**B**) The deubiquitinase OTUD7B maintains LSD1 stability by removing K63-linked ubiquitin (Ub) chains at K226/K277 (*72*). OTUD7B depletion leads to LSD1 dissociation from CoREST1/HDAC1/2, followed by p62-mediated proteasomal degradation, impairing metastasis. OTUD7B also stabilizes ERα, and its loss suppresses E2 signaling, indicating dual regulation of EMT and hormone response (*73*). (**C**) LSD1, CoREST1, HDAC1, and REST form a functional complex with ERα, supporting E2-dependent gene expression and proliferation. Upon E2 stimulation, LSD1 demethylates H3K9me2 at E2 target genes already preoccupied by LSD1, thereby promoting transcriptional activation by ERα. Knockdown or inhibition of LSD1 prevents H3K9me2 loss and impairs ERα chromatin binding and transcriptional activation (*77*). (**D**) Under long-term estrogen deprivation, luminal CD24^+^ ERα^+^ *TP53*^mut^ breast cancer cells undergo a phenotypic switch to CD44^+^ ERα^−^ basal-like states, resistant to antiestrogen therapy (*79*). This reprogramming involves a switch in CoREST complex partners—from ERα (parental) to cJUN-cBAF SWI/SNF (reprogrammed)—supporting survival and proliferation through repression of senescence and activation of MYC/E2F and EMT targets. These cells remain dependent on LSD1, as LSD1 knockout, but not catalytic inhibition, reverts them toward a parental-like phenotype and suppresses metastasis.

In breast cancer, LSD1 and CoREST1 expressions are highly correlated with Snail1 expression (*49*). LSD1 can associate with the SNAG domain of Snail1 to inhibit expression of important epithelial genes, thereby promoting cell migration (Fig. 3A). This LSD1/Snail1 interaction is further stabilized by CoREST1 and can be disrupted with LSD1 inhibitors (*49*). Indeed, LSD1 is an important regulator of EMT and cancer stem cell (CSC) gene programs in breast cancer (*69*). Its phosphorylation at serine 111 (LSD1-S111p) by protein kinase C–theta (PKC-θ) enhances its nuclear localization and reduces its H3K4 demethylase activity, thereby increasing the expression of EMT markers (Fig. 3A) (*69*). Interestingly, LSD1 and its S111p form were elevated in breast cancer cell lines that became resistant to docetaxel as well as in metastatic circulating patient tumor cells. Consistent with these data, iadademstat (ORY-1001), a potent and irreversible LSD1 inhibitor, abrogates the formation of CSC-derived mammospheres in CSC-enriched triple-negative breast tumor cells (*70*). LSD1 also has pathogenic roles in bone metastasis sites where it promotes production of formaldehyde, which is associated with bone metastasis pain (*71*).

The ubiquitination state of LSD1 has also been linked to breast cancer metastasis. High expression of OTU deubiquitinase 7B (OTUD7B) and LSD1 is associated with higher breast tumor grades (*72*). OTUD7B depletion results in an increase in K63-linked polyubiquitination of LSD1 at K226/K277, which results in its dissociation from CoREST1 and HDAC1/2 and its degradation through p62-mediated proteolysis (Fig. 3B). Degradation of LSD1, driven by OTUD7B depletion, impaired lung metastasis of LM2 breast cancer cells (*72*). Interestingly, OTUD7B is also known to stabilize estrogen receptor α (ERα), and its depletion results in degradation of ERα and suppression of 17β-estradiol (E2) signaling in breast cancer cells (Fig. 3B) (*73*). Almost two-thirds of breast tumors expresses or overexpresses ERα at the time of diagnosis. This results in enhanced estrogen signaling that can be targeted with antiestrogens, which are competitive inhibitors of the receptor. CoREST complex members LSD1, CoREST1, HDAC1, and REST all coimmunoprecipitate with ERα, and their knockdown inhibits ERα’s transcriptional activity and E2-dependent cell growth (Fig. 3C). Mechanistically, LSD1-mediated H3K9 demethylation takes place during transcriptional activation by nuclear hormone receptors such as ERα, estrogen-related receptor α, and the androgen receptor (AR) (*74*–*76*). In the case of ERα, E2 induces a marked reduction in H3K9me2 at regulatory regions of E2 target genes that are already preoccupied with LSD1 before E2 stimulation, suggesting that the reduction in H3K9me2 is not due to an increase in LSD1 recruitment but rather in its activity or activity of a newly recruited demethylase (*77*). The reduction in the H3K9me2 repressive mark is critical for E2-mediated ERα binding as knockdown or chemical inhibition of LSD1 abolished binding of the liganded receptor (Fig. 3C) (*77*). Moreover, E2 induced LSD1-dependent accumulation of nuclear foci of 8-oxo-guanine, a major DNA oxidation by-product, which triggered recruitment of 8-oxoguanine–DNA glycosylase 1 and topoisomerase IIβ to create chromatin conformational changes permissive for ERα binding (*77*). This is consistent with demethylation by LSD1 being an oxidative process that generates hydrogen peroxide (*78*). It is unclear how CoREST1 depletion compromises ERα’s transcriptional activity in the context of LSD1’s mechanism of action, although, as mentioned earlier, CoREST1 controls the demethylase activity and protein stability of LSD1 (*15*).

Important recent work indicates that *KDM1A*, *RCOR2*, and *HDAC2* are overexpressed in *TP53-*mutated breast tumors with higher expression in mutated tumors that are ERα^−^ (*79*). *TP53* mutations are present at a high frequency in more aggressive and less differentiated basal-like breast tumors (80%) compared to more differentiated luminal tumors (12 to 29%) (*80*). When luminal CD24^+^ ERα^+^ *TP*53^mut^ breast cancer cells are cultured under long-term estrogen deprivation, they transition into a “reprogrammed” CD44^+^ ERα^−^ basal-like phenotype resistant to antiestrogen therapy (*79*). Notably, the CoREST complex is a major driver of this transition whereby CoREST complex binding partners are switched from CoREST-ERα (parental state) to CoREST-cJUN-cBAF SWI/SNF (reprogrammed state) (Fig. 3D). The former reinforces E2 signaling, whereas the latter acts to repress senescence, autophagy, and apoptosis and induce MYC and E2F target genes. Reprogrammed cells with acquired resistance are sensitive to LSD1 knockout but not LSD1 chemical inhibition. In addition, LSD1 knockout was sufficient to revert reprogrammed cells back into a parental-like state and suppress metastasis in vivo, albeit without restoring expression of ERα. These data indicate that LSD1 inhibitors are unlikely to be effective in targeting endocrine resistance seen in 40 to 50% of patients with breast cancer (*81*, *82*), whereas CoREST degraders could have important clinical utility. It remains to be seen whether combined depletion of all CoREST complex members will elicit a more substantial suppression of this acquired resistance, and whether it will be sufficient to restore endocrine sensitivity by restoring ERα expression.

With regard to HDAC proteins, it is widely documented that HDAC inhibitors inhibit E2 signaling in breast cancer cells by inducing transcriptional repression of *ESR1*, the ERα-encoding gene, as well as ERα protein degradation (*83*–*88*). ZNF217, another member of the CoREST complex as mentioned previously, also interacts with and enhances recruitment of ERα to promote its transcriptional activity at E2 target genes (*89*). Importantly, ZNF217 overexpression confers resistance to tamoxifen, a widely used antiestrogen indicated for treatment of ERα-positive breast cancer (*89*).

In contrast to the overwhelming literature supporting an oncogenic role of the CoREST complex in breast cancer, a tumor suppressor role has also been reported. While LSD1 knockdown did substantially reduce proliferation of luminal-lineage breast cancer cells, it also increased their migration and invasion potential (*90*). LSD1 knockdown was also reported to enhance lung metastasis in the *MMTV-PyMT* luminal breast cancer mouse model (*90*). Analysis of the mutational landscape of LSD1 in the cancer genome atlas breast invasive carcinoma dataset revealed that it is <1% mutated. Of the four missense mutations identified, the R251Q mutation in the SWIRM domain enhanced breast tumor cell invasion and migration, thereby phenocopying LSD1 knockdown (*91*). The R251Q mutations disrupt the formation of the CoREST complex, leading to increased expression of the TRIM37 oncoprotein as well as EMT markers (*91*, *92*). Given that breast cancer is a highly heterogeneous disease with a great degree of lineage plasticity, we posit that knockdown of LSD1 could target more differentiated luminal cell populations for cell death and consequently enrich for a side population that is more susceptible to dedifferentiate into a stem-like/basal-like state. In light of its well-documented role in establishing differentiation blocks in leukemia, additional work is necessary to determine whether LSD1 and its partners also contribute to the differentiation blocks installed at different stages of mammary cell development as seen in the molecularly distinct subtypes of breast cancer. Additional work is also needed to examine how the CoREST complex regulates ERα transcriptional programs in E2-dependent breast tumors.

## CoREST TARGETING IN PROSTATE CANCER

**Fig. 4.**
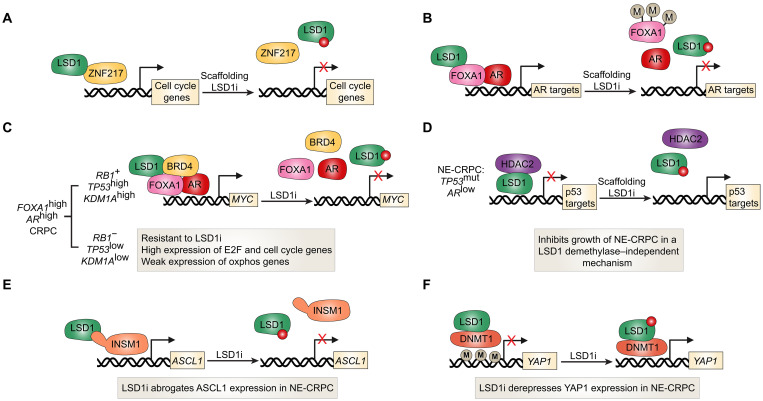
Diverse roles of LSD1 in regulating androgen signaling, lineage plasticity, and therapeutic response in prostate cancer. (**A**) In CRPC, LSD1 is highly expressed and partners with ZNF217 to drive expression of mitotic, cell cycle, and ESC genes. This interaction supports tumor growth and can be disrupted by the allosteric LSD1 inhibitor SP-2509, leading to CRPC growth inhibition independent of LSD1’s demethylase activity (*93*). (**B**) LSD1 promotes AR signaling by demethylating H3K9me1/2 and by modulating methylation of the pioneer transcription factor FOXA1, enhancing AR binding and transcription (*97*). (**C**) Single-cell RNA sequencing in FOXA1^high^ AR^high^ CRPC reveals molecular predictors of LSD1 inhibitor sensitivity (*94*). Responsive cells exhibit high expression of *TP53*, *RB1*, and *KDM1A*, while nonresponsive cells show low expression of these markers. In sensitive populations, LSD1 inhibitors suppress MYC expression and its targets by disrupting a complex of LSD1, BRD4, FOXA1, and AR. This effect underlies the observed synergy between LSD1 and BET inhibitors. (**D**) In AR^low^ NE-CRPC, which often arises through transdifferentiation and harbors TP53 mutations, LSD1 is overexpressed. Treatment with SP-2509 disrupts the LSD1-HDAC2 interaction, derepresses p53 target genes, and inhibits NE-CRPC growth in vitro and in vivo (*101*). These effects are not observed with catalytic inhibition or catalytically dead LSD1 mutants, suggesting a scaffolding role for LSD1 in NE-CRPC. (**E**) In NE-CRPC, LSD1 also interacts with INSM1, a SNAG domain transcription factor critical for neuronal gene expression. LSD1 inhibition disrupts this interaction, leading to down-regulation of the neuronal lineage factor ASCL1 and suppression of the neuroendocrine program (*102*). (**F**) LSD1 inhibition also results in derepression of YAP1, a key transcriptional regulator typically silenced in NE-CRPC. This occurs in part through reduced DNA methylation, restoring YAP1 expression and shifting the balance away from the neuroendocrine phenotype (*103*).

Aberrant AR expression and signaling are main drivers of prostate carcinogenesis. Androgen deprivation therapy (ADT) or hormone therapy remains the gold standard treatment for AR-positive prostate cancer. However, a large proportion of patients ultimately acquire resistance and progress to highly lethal metastatic castration-resistant prostate cancer (CRPC), which often maintains AR expression. LSD1 is highly expressed in CRPC where its interaction with ZNF217 is crucial for expression of a cluster of mitosis/cell cycle and embryonic stem cell (ESC) genes (Fig. 4A) (*93*). Disrupting the interaction between LSD1 and ZNF217 through SP-2509, an allosteric LSD1 inhibitor, inhibited the survival of CRPC cells in a mechanism that was independent of LSD1’s demethylase activity (Fig. 4A) (*93*). The mechanism of CRPC growth inhibition by LSD1 inhibitors appears to also rely on inhibition of MYC signaling (*94*).

As mentioned earlier, LSD1 can act as a coactivator to steroid hormone receptors by demethylating repressive H3K9me1/2 marks to activate transcription. Histone H3 phosphorylation at threonine 6 and 11, both of which are induced by AR activation, appears to favor LSD1 specificity toward H3K9me1/2 versus H3K4me1/2 (*95*, *96*). Another mechanism by which LSD1 can promote AR signaling is through modulating methylation of FOXA1, a pioneer transcription factor crucial for AR DNA binding and transcriptional activity (Fig. 4B) (*97*). Treatment with LSD1 inhibitors markedly abrogates AR and FOXA1 DNA binding and transcription of AR target genes resulting in CRPC growth inhibition in vivo (*97*). Notably, synergistic growth inhibition can be achieved in combination with antiandrogens (*97*). Similar results were also observed in prostate cancer models carrying the constitutively active AR-V7 variant, which is prevalent in approximately 18% of CRPC cases (*97*, *98*).

CRPC model cell lines can have a high degree of intrinsic heterogeneity. Single-cell RNA sequencing has revealed molecular determinants of sensitivity to LSD1 inhibitors in *AR*^high^/*FOXA1*^high^ CRPC cells (*94*). Cells that were *RB1*^positive^/*TP53*^high^/*KDM1A*^high^ were transcriptionally responsive to LSD1 inhibitors, whereas *RB1*^negative^/*TP53*^low^/*KDM1A*^low^ were nonresponsive (Fig. 4C). Importantly, exposure to LSD1 inhibitors caused a substantial reduction in expression of MYC and MYC targets in the responsive population but not the nonresponsive one. A complex comprising LSD1, BRD4, FOXA1, and AR was responsible for MYC expression in CRPC cells. LSD1 inhibitors abrogated genomic binding of BRD4, FOXA1, and AR (Fig. 4C). This correlated with synergistic growth inhibition seen with LSD1 inhibitors and BET inhibitors, which are known to strongly reduce MYC expression. AR-negative neuroendocrine CRPC (NE-CRPC) is a highly aggressive subtype of CRPC, which often harbors *TP53* mutations or deletions (~67% of cases), and develops through transdifferentiation of luminal adenocarcinoma cells (*99*, *100*). LSD1 expression was recently shown to be elevated in NE-CRPC compared to non-NE CRPC (*101*). Importantly, treatment with the allosteric LSD1 inhibitor SP-2509 disrupted the interaction between LSD1 and HDAC2 thereby derepressing p53 target genes, which in turn inhibited growth of NE-CRPC cells ex vivo and in vivo (Fig. 4D) (*101*). Notably, inhibition of LSD1 catalytic activity or expression of catalytically dead LSD1 mutant did not result in NE-CRPC growth inhibition (*101*). It remains to be seen whether LSD1, CoREST1, or other CoREST complex proteins are involved in the transition of luminal prostate cancer cells toward NE states and loss of AR. Indeed, more recent evidence indicates that LSD1 inhibition by bomedemstat suppresses the neuronal transcriptional program in NE-CRPC cells. This effect is mediated by the disruption of the interaction between LSD1 and INSM1, a SNAG domain transcription factor important for neuronal differentiation (Fig. 4E). As a result, ASCL1, a key neuronal lineage driver, is down-regulated (*102*). In parallel, YAP1 is derepressed—partly through reduced DNA methylation—restoring its expression, which is typically silenced in both human and mouse NE-CRPC (Fig. 4F) (*102*, *103*).

Similar to breast cancer where they inhibit E2 signaling, HDAC inhibitors or HDAC1/3 knockdown suppresses AR signaling in prostate cancer cells by preventing recruitment of AR coactivators, SRC1 and p300 to AR target genes (*104*). The contribution of CoREST1 in pathogenesis of CRPC is not known although CoREST1 knockdown, similar to LSD1 inhibition, reduces induction of AR target genes in the presence of androgens (*105*). In addition, CoREST1 coimmunoprecipitates with FOXA1 and AR and BRD4, which suggests that it may also be implicated in regulating MYC expression in CRPC (*94*). Collectively, these data support a central role of LSD1, ZNF217, and HDAC proteins in prostate cancer pathogenesis, whose targeting may allow circumvention of ADT resistance.

## CoREST TARGETING IN LUNG CANCER

**Fig. 5.**
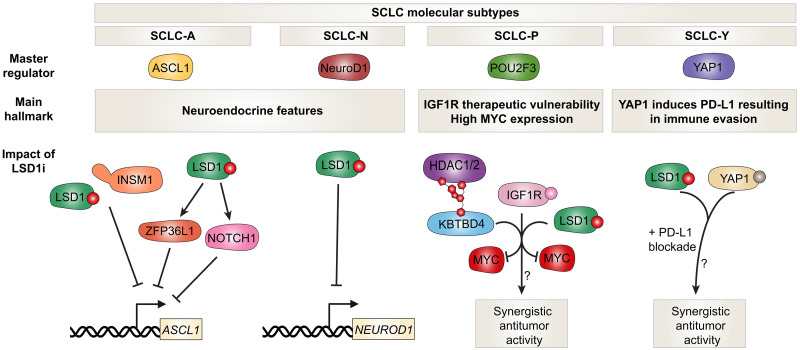
LSD1 inhibition disrupts neuroendocrine transcriptional programs in SCLC. Four molecular subtypes of SCLC have been defined by expression of lineage-specific transcription factors: ASCL1 (SCLC-A), NeuroD1 (SCLC-N), POU2F3 (SCLC-P), and YAP1 (SCLC-Y) (*109*). SCLC-A and SCLC-N exhibit high expression of neuroendocrine markers, driven by ASCL1 and NeuroD1, which are essential for SCLC growth and survival. SCLC-P tumors express tuft cell markers and are regulated by POU2F3, with a reported vulnerability to IGF1R inhibition. SCLC-Y tumors, which show the lowest neuroendocrine gene expression, have the worst prognosis and are characterized by YAP1-driven immune evasion via PD-L1 up-regulation. LSD1 inhibitors block the LSD1-INSM1 interaction, suppressing neuronal differentiation programs and substantially reducing ASCL1 and NeuroD1 expression, leading to antitumor activity in both ex vivo and in vivo models (*115*). LSD1 inhibition also induces NOTCH1 expression and activates NOTCH signaling, which contributes to neuroendocrine gene suppression through down-regulation of ASCL1 (*116*). In parallel, LSD1 inhibition up-regulates the mRNA binding protein ZFP36L1, which reinforces the suppression of ASCL1 and neuroendocrine fate (*117*). Therapeutically, LSD1 inhibitors may synergize with IGF1R pathway inhibitors in SCLC-P, paralleling observations in AML (*121*). UM171-mediated activation of KBTBD4 suppresses MYC expression (*124*), which is a hallmark of the SCLC-P subtype. Furthermore, LSD1 inhibition has been shown to enhance response to anti–PD-L1 immunotherapy and exhibits additive antiproliferative effects when combined with YAP inhibitors in other tumor models (*122*, *123*). Whether these combinatorial effects extend to YAP1-driven SCLC-Y remains to be determined.

Lung cancer continues to be the leading cause of cancer death in the US and Canada (*106*, *107*). Non–small cell lung cancer (NSCLC) is the most common type of lung cancer, whereas small cell lung cancer (SCLC) accounts for ~15% of cases (*108*). SCLC is highly aggressive with NE features, more rapid doubling time, and earlier occurrence of metastasis (*108*). While most patients initially respond to chemotherapy, most of them eventually die from disease recurrence (*108*). Four molecular subtypes of SCLC have been described on the basis of differential expression of four master transcription factors: ASCL1 (SCLC-A), NeuroD1 (SCLC-N), POU2F3 (SCLC-P), and YAP1 (SCLC-Y) (Fig. 5) (*109*). NE markers are highly expressed in SCLC-A and SCLC-N, whereas SCLC-P and SCLC-Y have a low NE transcriptional program (Fig. 5) (*109*). ASCL1 and NeuroD1 regulate NE differentiation in the lung and are crucial for SCLC pathogenesis and survival (*110*). POU2F3 is a master regulator of SCLC-P tumors, which express chemosensory markers found in tuft cells in the lung (*111*). Notably, SCLC-P cell lines have an IGF1R therapeutic vulnerability. YAP1 induces expression of PD-L1 causing inhibition of T cell infiltration in SCLC-Y tumors, which have the worst prognosis among all four SCLC subtypes (*112*).

There is growing evidence of LSD1 being a therapeutic target in SCLC. LSD1 is highly expressed in SCLC cell lines and primary specimens compared to other lung cancer subtypes (*113*). Indeed, GSK2879552, a potent LSD1 inhibitor, showed antiproliferative activity in a panel of SCLC cell lines (*114*). DNA hypomethylation of a signature probe set was predictive of sensitivity to LSD1 inhibition, whereas MYC-amplified/high SCLC cells were resistant (*114*). The LSD1 inhibitor T-3775440 abrogates the interaction between LSD1 and INSM1, which is important for neuronal differentiation and survival of SCLC cells (Fig. 5) (*115*). Notably, T-3775440 also abrogates expression of ASCL1 and NeuroD1, whose loss contributes to antitumor activity both ex vivo and in vivo (Fig. 5) (*115*). Antitumor activity of LSD1 inhibitors in SCLC is also attributed to induction of NOTCH1 expression and subsequent activation of NOTCH signaling, which contributes to strong ASCL1 down-regulation and repression of NE genes (Fig. 5) (*116*). The mechanism by which LSD1 inhibitors block NE differentiation in SCLC has also been linked to increased expression of the mRNA binding protein ZFP36L1, which in turn also contributes to ASCL1 suppression (Fig. 5) (*117*). ZFP36L1 can bind and destabilize SOX2 and INSM1 mRNAs, which are necessary for maintenance of the NE phenotype (*117*). Importantly, and similar to SCLC, sensitivity of a panel of AML cell lines to iadademstat was dependent on induction of ZFP36L1, whose knockout conferred resistance to LSD1 inhibition (*117*). Indeed, NE ASCL1^high^ or NeuroD1^high^ SCLC cell lines were sensitive to GSK690, an LSD1 inhibitor, whereas cell lines that were mesenchymal in nature were intrinsically resistant (*113*). Notably, only partial responses to LSD1 inhibitors were seen in sensitive SCLC cell lines, which were attributed to TEAD4 activation–acquired resistance that could be alleviated with TEAD inhibitor cotreatment (*113*). Whether targeting catalytic and/or scaffolding properties of LSD1 is driving antiproliferative activity in SCLC requires further investigation. Notably though, GFI1B and INSM1-expressing SCLC cells were not sensitive to T-448, a LSD1 inhibitor that does not disrupt LSD1 interactions with SNAG domain factors (*113*).

A transcript variant of LSD1 referred to as LSD1+8a is generated through alternative RNA splicing and inclusion of the E8a exon in the LSD1 mature mRNA. LSD1+8a expression was originally described to be restricted to cells of neuronal lineage with an important role in cortical neuron differentiation (*118*). The LSD1+8a isoform lacks capacity to demethylate H3K4 but is selective toward H3K9 demethylation to promote neuronal differentiation (*119*). Interestingly, the LSD1+8a isoform is expressed in SCLC cell lines where it contributes to expression of NE genes (*120*). LSD1+8a^high^ SCLC cell lines were resistant to LSD1 inhibitors and chemotherapeutic agents, but they were sensitive to specific LSD1+8a transcript knockdown (*120*).

Collectively, the aforementioned data indicates that LSD1 provides a promising therapeutic vulnerability in NE SCLC tumors. LSD1 inhibition has been reported to synergize with insulin/IGF1R signaling inhibition for suppression of AML cell growth (*121*). It remains to be seen whether such a synergy also takes place in non-NE SCLC-P tumor subtypes (Fig. 5). In addition, LSD1 inhibitors can sensitize SCLC cells to anti–PD-L1 blockade and have shown an additive antiproliferative effect when combined with YAP inhibitors in oral squamous cell carcinoma cells (*122*, *123*). Whether this applies in YAP1-driven SCLC requires further elucidation (Fig. 5). The exact contribution of CoREST1 and HDAC1/2 in the context of CoREST complexes in regulating NE differentiation in SCLC is not known. It is conceivable that NE SCLC tumors sensitive to LSD1 inhibitors would be equally as sensitive or more to CoREST complex degraders. As mentioned earlier, elevated MYC expression correlates with resistance to LSD1 inhibitors. Additional work is needed to determine whether SCLC-P tumor subtypes, which express the highest levels of MYC across all SCLC subtypes, would benefit from KBTBD4 activation through UM171, which not only degrades CoREST complex proteins but also suppresses MYC expression (*109*, *114*, *124*).

## CoREST TARGETING IN NERVOUS SYSTEM CANCERS

Whereas neuroblastomas (NB) are tumors that arise in the peripheral nervous system, gliomas and medulloblastomas (MB) arise in the brain. NB is the most common cancer in infants and most common extracranial solid tumor in children (*125*). It is thought to arise from abnormal development of sympathoadrenal precursors in the neural crest, which is accompanied with a block in neuronal differentiation. 13-cis-retinoic acid treatment can inhibit NB cell proliferation and induce cell differentiation (*126*). In the clinic, pediatric high-risk NB standard treatment includes chemotherapy and autologous stem cell transplantation followed by anti-GD2 therapy combined with granulocyte-macrophage colony-stimulating factor, interleukin-2 (IL-2), and 13-cis-retinoic acid.

**Fig. 6.**
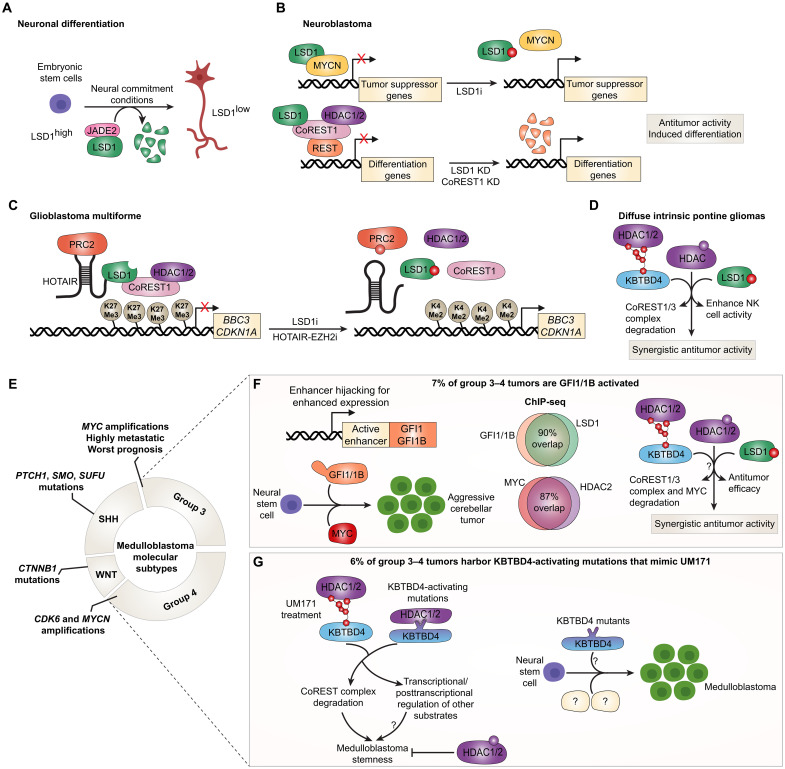
Targeting the CoREST complex in neural cancers. (**A**) During neural commitment, LSD1 is degraded by the E3 ligase Jade-2, promoting differentiation (*130*). (**B**) In NB, LSD1 cooperates with MYCN to repress tumor suppressor genes (*CDKN1A*, *CLU*, and *NDRG1*), enhancing proliferation and invasion (*131*, *132*). The LSD1 inhibitor SP-2509 suppresses MYCN targets and induces differentiation (*133*). LSD1 or CoREST1 knockdown also reduces REST, alleviating differentiation blocks (*144*, *145*). (**C**) In glioblastoma, the long noncoding RNA HOTAIR scaffolds PRC2 and CoREST complexes to drive gene repression. Combined disruption of HOTAIR-EZH2 interaction and LSD1 inhibition derepresses PUMA and p21, enhancing tumor suppression (*150*). (**D**) In diffuse intrinsic pontine glioma, dual inhibition of LSD1 and HDACs with corin increases histone methylation and acetylation, induces differentiation and suppresses tumor growth (*155*). LSD1 inhibition also enhances innate immune signaling and NK cell–mediated tumor clearance (*156*). UM171-mediated LSD1 degradation synergizes with HDAC inhibitors, underscoring LSD1’s scaffolding role (*157*). (**E**) MB subgroups (WNT, SHH, group 3, and group 4) display distinct genetic drivers (*158*). Group 3/4 tumors frequently exhibit MYC, MYCN, or CDK6 amplifications, while WNT and SHH tumors harbor mutations in *CTNNB1* or the SHH pathway, respectively. (**F**) In group 3/4 MB, enhancer hijacking causes GFI1/GFI1B overexpression (*159*). Coexpression of MYC and GFI1/1B together but not separately drives aggressive tumors, reliant on LSD1-GFI1 and MYC-HDAC2 co-occupancy (*159*, *160*, *162*). LSD1 or HDAC2 inhibition disrupts this axis, suppressing tumor growth. (**G**) Recurrent KBTBD4 mutations in group 3/4 MB and pineal tumors, namely, KBTBD4^R313PRR^ and KBTBD4^P311PP^, mimic UM171, promoting CoREST complex degradation and tumor stemness (*27*, *164*, *166*). Although their full oncogenic role remains unclear, targeting the CoREST complex in these tumors will not be beneficial. In contrast, MYC- and GFI1-driven tumors display CoREST dependency, suggesting a therapeutic vulnerability. ChIP-seq, chromatin immunoprecipitation sequencing.

MYCN gene amplifications are highly prevalent in high-risk NB and are associated with poor overall survival rates (*127*, *128*). Furthermore, LSD1 expression is inversely correlated with differentiation status of NB and overall survival (*129*). LSD1 protein levels markedly decline because of degradation by the ubiquitin ligase Jade-2 when mouse ESCs are cultured under neural commitment conditions (Fig. 6A) (*130*). Neuronal differentiation of ESCs under these culture conditions was decelerated upon knockdown of Jade-2, which could be reversed when combined with knockdown of LSD1, thus pointing to a central antibraking role of LSD1 in mouse neurogenesis. Importantly, while Jade-2 coimmunoprecipitates with the CoREST complex, it promoted NB cell differentiation through specifically enhancing LSD1 ubiquitination and degradation. Knockdown of LSD1 or its chemical inhibition also abolishes NB growth both ex vivo and in vivo (*129*). Moreover, LSD1 can associate with MYCN on promoters of tumor suppressor genes such as CDKN1A (p21) and CLU (clusterin) to suppress their expression (Fig. 6B) (*131*). LSD1 and MYCN also cooperate to suppress expression of NDRG1, which results in enhanced motility and invasiveness of NB cells (*132*). Expectedly, SP-2509 represses the MYCN gene signature and induces differentiation pathway genes in NB cells (*133*). These data are consistent with TCP synergizing with 10058-F4, a MYC/MYCN inhibitor, for NB growth inhibition (*131*). TRIM24 is an E3 ligase highly expressed in MYCN-amplified NB (*134*). TRIM24 depletion inhibits NB cell proliferation and induces cell differentiation through disrupting CoREST complex formation without altering CoREST complex protein expression (*134*). Of note, TRIM24 has known oncogenic roles in ERα and AR signaling in breast and prostate cancer, respectively, although it remains unclear whether the CoREST complex plays a part in these roles (*135*, *136*).

As discussed so far, the CoREST complex coopts different transcription factors or cofactors (REST, GFI1, ERα, AR, INSM1, and ZNF217) to elicit tissue-specific transcriptional responses. In human and mouse NB cells, the canonical CoREST complex also associates with myelin transcription factor 1 (MYT1), a neural-specific zinc finger transcription factor, to directly regulate common neural-specific target genes (*137*, *138*). In human NB, MYT1 is overexpressed and associates with shorter overall survival rates and poor differentiation (*138*). Depletion of LSD1 or its catalytic inhibition by iadademstat reduces expression of MYT1 and enhances neurite outgrowth in NB cells in the presence of 13-cis-retinoic acid (*138*). Similarly, knockdown or chemical inhibition of HDAC1 and HDAC2 induces NB cell differentiation and inhibits NB cell viability (*139*, *140*). As mentioned earlier, the CoREST complex also associates with REST, which is a master negative regulator of NSC differentiation that serves to prevent precocious neuronal differentiation (*141*). REST expression is elevated in NB tumors (*142*). Consistent with its low expression in mature neurons compared to NSCs, REST expression is also reduced upon differentiation by 13-cis-retinoic acid in NB cells (*142*). Knockdown of USP3, a deubiquitinase that regulates REST protein stability, enhanced 13-cis-retinoic acid–mediated NB cell differentiation and counteracted REST-mediated tumorigenesis in vivo (*143*). Notably, knockdown of LSD1 or CoREST1 results in reduced expression of REST (Fig. 6B) (*144*, *145*). Together, these data indicate that targeting the CoREST complex in NB may block both REST-dependent and REST-independent pathogenic mechanisms.

Glioblastoma multiforme (GBM) is the most common and malignant type of brain cancer accounting for ~45% of all cases, with a median survival of 15 months (*146*). Knockdown of REST in GBM stem cells impaired their self-renewal capacity, reduced tumor invasiveness, and increased survival in transplanted mice (*147*). The long intergenic noncoding RNA HOTAIR was first reported to act as a modular scaffold for both the PRC2 polycomb complex and the CoREST1 (LSD1/CoREST1/REST) complex, to elicit coordinated H3K27 methylation and H3K4 demethylation (Fig. 6C) (*148*). HOTAIR is necessary for GBM cell growth (*149*). In addition, combined treatment with a HOTAIR-EZH2 disrupting agent and an LSD1 inhibitor resulted in more profound tumor inhibition in vivo compared to either treatment alone through derepression of PUMA and p21 expression (Fig. 6C) (*150*). LSD1 knockdown or inhibition in GBM stem cells was also reported to diminish their capacity to undergo homologous recombination and nonhomologous end-joining DNA repair thereby enhancing efficacy of temozolomide, an adjuvant chemotherapeutic agent (*151*).

Diffuse intrinsic pontine gliomas (DIPG) are a type of high-grade glioma prevalent in children and are primarily driven by epigenetic dysregulation. These tumors harbor H3K27M mutations in 78% of the cases, which are known to contribute strongly to disease pathogenesis by markedly reducing H3K27me3 (*152*–*154*). Recent work demonstrates that LSD1 knockout sensitizes DIPG cells to HDAC inhibitors (Fig. 6D) (*155*). Treatment with corin, which is a bifunctional LSD1 and HDAC inhibitor, increased H3K27me3, H3K27ac, and H3K4me1, enhanced cell differentiation and apoptosis, and suppressed DIPG growth ex vivo and in vivo (*155*). Remarkably, LSD1 inhibition in DIPG cells increases expression of innate immune receptors that enhance natural killer (NK) cell activity (*156*). DIPG-implanted mice exposed to GSK-LSD1 intraperitoneally and human ex vivo–expanded NK cells intracranially exhibited a 43% reduction in tumor burden versus 23% when exposed to NK cells alone (*156*). CRISPR screens in DIPG cell lines have also identified LSD1 knockout to synergize with the HDAC inhibitor panobinostat, a phenotype that could not be seen with LSD1 inhibitors (*157*). Indeed, similar to the screen results, UM171-mediated degradation of LSD1 synergized with panobinostat for DIPG cell growth inhibition (Fig. 6D), which highlights the importance of the scaffolding function of LSD1 (*157*).

MBs are the most common malignant brain tumors in children. Four molecular subgroups of MB have been described: WNT, SHH, group 3, and group 4 (Fig. 6E) (*158*). The first two groups are characterized by wnt and sonic hedgehog signaling mutations and have better prognosis, whereas group 3 is characterized by MYC amplifications and group 4 have CDK6 and MYCN amplifications (*158*). Group 3 is highly metastatic and has the worst prognosis across all subgroups. Importantly, Northcott *et al.* (*159*) identified a series of spatially clustered somatic structural variants at the 9q34 locus in ~7% of group 3 and group 4 tumors. These structural variants caused enhanced expression of GFI1 or GFI1B in a mutually exclusive manner through enhancer hijacking (Fig. 6F) (*159*). Overexpression of MYC and GFI1/GFI1B together but not separately in neural mouse stem cells resulted in highly aggressive cerebellar tumors with poor neuronal differentiation (Fig. 6F) (*159*, *160*). However, overexpression of MYC with a GFI1 P2A mutant, which disrupts the latter’s SNAG domain, in neural progenitors prevented tumor formation in mice (*160*). GFI1 coimmunoprecipitated with LSD1 and CoREST1 in these tumors, and up to 90% of LSD1 and GFI1 genomic binding sites were overlapping (Fig. 6F) (*160*). In line with this, growth of these GFI1/MYC-dependent MB tumors was abrogated upon knockdown or chemical inhibition of LSD1 (*160*). MYC-dependent MB tumors also rely on HDAC2 for survival (*161*, *162*). A notable 87% of MYC genomic binding sites overlap with HDAC2 binding in primary group 3 MB tumors (Fig. 6F) (*162*). Entinostat, a class I HDAC inhibitor with preferential activity toward HDAC1 (versus HDAC2-3), abrogated MYC transcriptional activity (Fig. 6F) (*162*). In addition, pan-specific HDAC inhibitors abrogated tumor growth in a MYC-driven group 3 MB mouse model in part through enhancing expression of FOXO1 (*163*).

Recently, recurrent in-frame hotspot mutations in the second kelch domain repeat of KBTBD4, the target of UM171 as mentioned earlier, were identified in 6% of group 3 and group 4 MB (Fig. 6G) (*27*, *164*). Of note, these mutations were mutually exclusive with other driver events including MYC and MYCN amplifications. Hotspot mutations in *KBTBD4* were also identified in patients with pineal parenchymal tumor of intermediate differentiation, which arises from the pineal gland in the brain midline (*165*). These neomorphic KBTBD4 mutations, namely, KBTBD4^R313PRR^ and KBTBD4^P311PP^, phenocopy the mechanism of action of UM171 and enhance the recognition, ubiquitination, and degradation of the CoREST complex (Fig. 6G) (*27*, *166*). Consistent with the stem cell–enhancing properties of UM171 in the blood system (*26*, *27*), forced expression of these activating KBTBD4 mutants or LSD1 inhibitor treatment also enhanced stemness of MB cells (*166*). However, an important question that remains unresolved is whether expressing neomorphic KBTBD4 mutants in mouse NSCs will be sufficient to generate group 3/4 MB tumors in vivo, or whether additional KBTBD4-converging driver events are necessary (Fig. 6G). The exact contribution of CoREST degradation and CoREST-independent mechanisms in pathogenesis of KBTBD4^mutant^ MB tumors is also unclear. Overall, while KBTBD4^mutant^-driven group 3 and 4 MB may not benefit from a CoREST complex–targeting therapy, it remains clear, given the above, that GFI1/1B- or MYC-driven MB tumors have a CoREST complex vulnerability that merits further investigation.

## CoREST TARGETING IN NEURODEGENERATIVE DISEASES

**Fig. 7.**
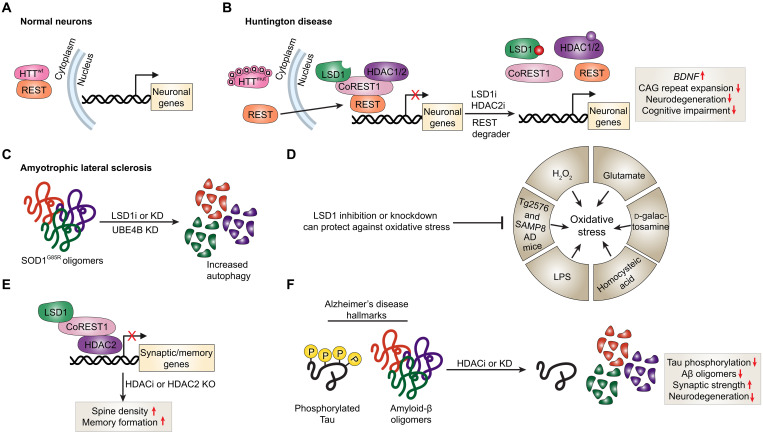
Targeting the CoREST complex confers neuroprotective effects in neurodegenerative diseases. (**A**) In healthy neurons, REST is sequestered in the cytoplasm by wild-type huntingtin (HTT), which prevents it from suppressing neuronal gene expression (*168*). (**B**) In HD, mutant HTT fails to bind REST, allowing its nuclear translocation and repression of neuronal genes such as *BDNF* (*168*). REST inhibition via X5050, C91, or the LSD1 inhibitor vafidemstat rescues *BDNF* expression and cognitive deficits in HD models (*172*, *173*). HDAC2 knockout or inhibition also reduces CAG repeat expansion and neurodegeneration (*176*–*180*). (**C**) In amyotrophic lateral sclerosis (ALS), knockdown of LSD1 and UBE4B suppresses *SOD1*^G85R^ proteotoxicity by enhancing autophagy and oligomer proteasomal clearance. LSD1 inhibition induces autophagy through sestrin 2 up-regulation and mTORC1 inhibition (*181*, *182*). (**D**) LSD1 inhibition protects against oxidative stress in neurons and nonneuronal tissues. Compounds such as TCP, bizine, TAK-418, and vafidemstat improve memory and social behavior and reduce inflammation in models of autism, AD, and ischemia-reperfusion injury (*183*–*190*). (**E**) In AD, HDAC2 negatively regulates synaptic plasticity, memory, and learning. HDAC2 inhibition or knockout restores synaptic density and memory function (*197*, *198*). (**F**) Class II HDAC inhibitors reduce Aβ and tau pathology, enhancing expression of mmp2 and improving cognition in AD models (*199*). In the mouse brain, CoREST1 preferentially associates with HDAC2 (*198*), and compounds like Rodin-A, which selectively inhibit HDAC1/2 within LSD1-containing complexes, enhance synaptic strength and memory (*200*), highlighting the CoREST complex as a potential therapeutic target. LPS, lipopolysaccharide.

Huntington disease (HD) is an autosomal dominant neurodegenerative disorder caused by CAG repeat expansions in the huntingtin gene *HTT* resulting in a polyglutamine tail at the N terminus (*167*). REST is normally sequestered into the cytoplasm in neurons by interacting with huntingtin (Fig. 7A) (*168*). However, in HD, *HTT* mutations abrogate REST binding to huntingtin resulting in REST nuclear translocation and inhibition of neuronal target genes (Fig. 7B) (*168*). One of these targets is *BDNF*, which is a neurotrophic factor critical for survival of striatal neurons that are lost in HD (*169*). Indeed, *BDNF* expression is markedly reduced in human HD cortices (*170*). Interestingly, the bifunctional miR-9/miR-9* miRNA is down-regulated in HD and has been reported to inhibit expression of REST and CoREST1 (*171*). Intraventricular delivery of X5050, a small-molecule REST degrader, strongly enhanced *BDNF* expression in the prefrontal cortices of mice with quinolinic acid–induced brain striatal lesions (Fig. 7B) (*172*). Similar results were also observed in NSCs derived from induced pluripotent stem cells from a patient with HD (*172*). C91, a quinolone-like compound, also inhibited REST activity by enhancing translocation of SIN3A to the cytoplasm in HD cells thereby increasing BDNF expression. Notably, vafidemstat (ORY-2001), a brain-penetrable LSD1 and monoamine oxidase-B inhibitor, was reported to stop/prevent the development of cognitive impairment in the R6/1 HD mouse model through counteracting transcriptional imbalances caused by REST nuclear translocation in seen in HD (Fig. 7B) (*173*). The CAG repeats in the *HTT* gene undergo further expansion in a time-dependent and cell type–dependent manner (*174*). Medium-spiny striatal neurons are particularly highly sensitive to this mutation (*175*). Knockout of HDAC2 in medium-spiny striatal neurons in the *Htt*^Q111^ HD mouse model reduced CAG repeat expansions over time and the intensity of diffuse nuclear mutant huntingtin (*176*). Consistent with this, a number of HDAC inhibitors could arrest neurodegeneration and ameliorate motor deficits in HD mouse models (Fig. 7B) (*177*–*180*).

Proteotoxic stress is a primary pathogenesis hallmark of neurodegenerative diseases. Genetic studies conducted in the roundworm *Caenorhabditis elegans* revealed that combined suppression of ufd-2 and spr-5, whose human orthologs are *UBE4B* and *KDM1A*, respectively, resulted in robust suppression of neurotoxicity driven by the amyotrophic lateral sclerosis (ALS)–linked *SOD1*^G85R^ mutation (*181*). The latter mutation causes misfolding and accumulation of soluble SOD1 oligomers and insoluble aggregates, which are directly linked to neurodegeneration (Fig. 7C). Knockdown of *UBE4B* and *KDM1A* in HEK293 cells expressing mutant SOD1 also resulted in clearance of misfolded SOD1 in a mechanism that involved increased proteasomal activity and autophagy and also relied on p53 activation (Fig. 7C) (*181*). Indeed, chemical inhibition of LSD1 or its knockdown also activates autophagy in NB cells by inducing expression of sestrin 2, which inhibits mTORC1 activity (*182*). Whether this mechanism of action holds true in affected neurons in ALS or other neurodegenerative diseases remains to be seen.

Consistent with these data, chemical targeting of LSD1 can be protective in certain cellular stress contexts that, in some cases, extend beyond the nervous system (Fig. 7D). For instance, TCP protects retinal ganglion cells against glutamate neurotoxicity and hydrogen peroxide–induced oxidative stress (*183*). LSD1 inhibition through bizine, a phenelzine analog, also protects primary cortical neurons against oxidative stress induced by homocysteic acid (*184*). TAK-418 and T-448, two LSD1 inhibitors, improve social and cognitive deficits in mouse models of autism spectrum disorders (*185*, *186*). TAK-418 also improved recognition memory in aged mice and the Tg2576 AD mouse model (*187*). LSD1 chemical inhibition through vafidemstat also rescued memory loss and corrected behavioral alterations in the senescence accelerated mouse prone 8 model of AD and accelerated aging (*188*). Mechanistically, vafidemstat induced expression of cognitive function genes and reduced those involved in neuroinflammation (*188*). The LSD1 inhibitor GSK-LSD1 was also been shown to counteract oxidative stress and acute liver injury caused by lipopolysaccharide/d-galactosamine exposure in mice (*189*). TCP or LSD1 knockdown also protects against oxidative stress and ferroptosis induced by renal ischemia reperfusion injury both in vivo and ex vivo through diminished Toll-like receptor 4/NOX4 signaling (*190*).

Alzheimer disease (AD) pathogenesis is primarily linked to formation of aggregates of extracellular amyloid-β (Aβ) plaques and intracellular neurofibrillary tangles of hyperphosphorylated microtubule–associated tau proteins (*191*). Neuroinflammation is also a critical contributing factor to AD pathogenesis. Aggregates of plaques and neurofibrillary tangles bind to cell surface receptors on microglia and astroglia resulting in an innate immune response and inflammatory cytokine release (*192*). Inflammatory cytokines can induce expression of inducible nitric oxide synthase in microglia and astroglia causing an increase in neurotoxic nitric oxide (*193*). Interestingly, administration of entinostat to cerebral amyloidosis transgenic APP/PS1 mice substantially reduced microglial activation, enhanced clearance of Aβ plaques, and importantly, reduced neuroinflammation ex vivo (*194*). There is a plethora of evidence of therapeutic benefit of HDAC inhibitors in neurodegenerative diseases, which are largely characterized by memory and learning deficits. Administration of sodium butyrate in the CK-p25 transgenic neurodegeneration mouse model increased expression of synaptic proteins, ameliorated learning deficits, and restored access to long-term memories (*195*). Similar results have also been observed with trichostatin A, a potent class I HDAC inhibitor, whereby memory consolidation was enhanced in a mechanism dependent on CREB/CBP signaling (*196*). Subsequent studies revealed a critical negative role of HDAC2 in regulating learning and memory processes in mice (Fig. 7E) (*197*, *198*). HDAC2 expression is elevated in postmortem brain tissue of patients with AD, in cell lines upon exposure to AD modeling neurotoxins, and in the CK-p25 and 5xFAD neurodegeneration mouse models (*197*). Importantly, mice overexpressing HDAC2, but not HDAC1, in the neuronal compartment exhibited reduced synaptic numbers and memory and learning impairment that can be alleviated with HDAC inhibitors (*198*). Conversely, HDAC2 knockout or treatment with HDAC inhibitors derepressed genes related to synaptic plasticity and memory resulting in enhanced spine density and memory formation (*197*, *198*). Notably, class II HDACs appear to also play a role in AD pathogenesis as inhibitors of this class can reduce Aβ and tau phosphorylation levels and rescue learning and memory deficits in the hAPP 3xTg AD mouse model by enhancing expression of the Aβ degradation enzyme mmp2 (Fig. 7F) (*199*). It is not known whether HDAC2 negatively regulates mouse cognitive functions as part of the CoREST complex or other repressive complexes. In the mouse brain, CoREST1 preferentially associates with HDAC2, as the latter is more abundantly expressed compared to HDAC1 (*198*). Rodin-A, a molecule that selectively inhibits HDAC1/2 associated with LSD1-immunoprecipitated complexes, enhanced spine density and expression of synaptic markers in mice (*200*). Importantly, Rodin-A also improved long-term potentiation of synaptic strength, which is otherwise impaired in the 5xFAD neurodegeneration mouse model of amyloid pathology (*200*). Collectively, these data indicate that targeting HDAC2 associated with the CoREST complex has therapeutic benefit in neurodegenerative mouse models. Whether targeting CoREST1 separately or together with LSD1 and HDAC2 would generate similar or additional prosynaptic benefit to HDAC2 targeting requires further elucidation.

## CoREST TARGETING ENHANCES ANTITUMOR IMMUNITY

**Fig. 8.**
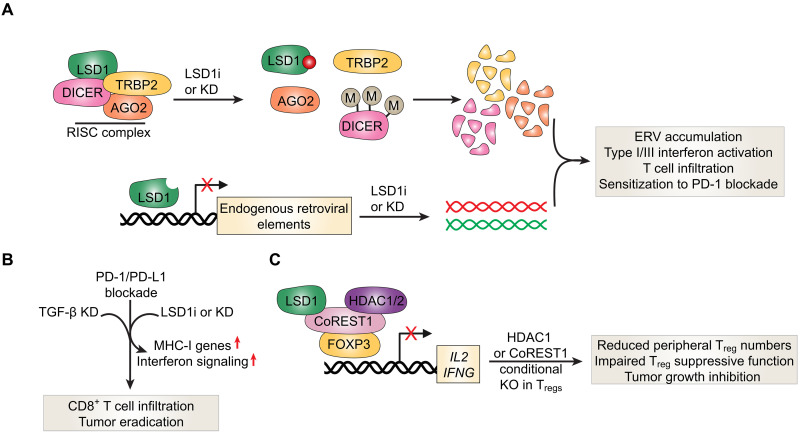
Targeting the CoREST complex enhances antitumor immunity and overcomes immune resistance. (**A**) LSD1 inhibition or knockdown leads to derepression of endogenous retroviral elements and suppression of RNA silencing complex proteins, resulting in dsRNA accumulation, activation of type I/III IFNs, and sensitization to PD-1 blockade (*201*). (**B**) In melanoma, LSD1 knockout sensitizes tumors to PD-1 blockade by enhancing CD4^+^ and CD8^+^ T cell infiltration, although tumor eradication is limited by TGF-β–mediated immune cell exclusion from the parenchyma (*201*–*203*). Triple targeting of LSD1, TGF-β, and PD-1 yields robust CD8^+^ T cell infiltration and tumor clearance. In SCLC, LSD1 inhibition up-regulates MHC-I and antigen presentation genes, sensitizing cells to T cell cytolysis and enhancing PD-L1 blockade efficacy (*122*). (**C**) CoREST complex targeting in T_regs_ via LSD1/HDAC dual inhibitors or conditional *RCOR1* or *HDAC1* deletion impairs suppressive function, reduces peripheral T_regs_, and enhances antitumor immunity (*210*). The CoREST complex binds Foxp3, maintaining T_reg_ identity by repressing IL-2 and IFN-γ. In contrast, HDAC2 deletion enhances T_reg_ function, highlighting functional divergence among HDACs (*210*, *211*).

It is becoming increasingly evident that targeting the CoREST complex extends beyond suppressing autonomous cancer cell growth to also promoting adaptive antitumor immunity. LSD1 inhibition or knockdown causes reactivation of endogenous retroviral element transcripts and reduction of expression of RNA-induced silencing complex proteins (Fig. 8A) (*201*). This resulted in double-stranded RNA (dsRNA) formation and accumulation, which triggered type I/III interferon (IFN) activation and cancer growth inhibition (Fig. 8A) (*201*). Importantly, LSD1 knockout in PD-1 blockade–resistant melanoma cells increased CD4^+^ and CD8^+^ T cell infiltration in vivo and sensitized cells to PD-1 blockade, although complete tumor eradication was not observed (*201*). The latter was attributed to increased expression of TGF-β upon LSD1 knockout, which is known to inhibit response to anti–PD-L1 antibodies through exclusion of CD8^+^ T cells from the tumor parenchyma (*202*, *203*). Indeed, simultaneous depletion of LSD1 and TGF-β together with PD-1 blockade resulted in increased CD8^+^ T cell infiltration and tumor eradication in vivo (Fig. 8B) (*202*).

In SCLC, *KDM1A* expression is inversely correlated with expression of major histocompatibility complex class I (MHC-I) as well as antigen presentation pathway genes (*122*). Iadademstat treatment rescued expression of MHC-I/antigen presentation pathway genes and increased IFN signaling in several SCLC cell lines only after at least 7 days of exposure (Fig. 8B) (*122*). Similar results were also obtained with LSD1 depletion. Consistent with the aforementioned, LSD1 inhibition also sensitizes SCLC cells to MHC-I–restricted T cell cytolysis and results in an augmented antitumor response in combination with PD-L1 blockade therapy as mentioned earlier (*122*). In NSCLC, the E3 ligase TRIM35 acts as a tumor suppressor by inhibiting LSD1’s demethylase activity through K63 ubiquitination at LSD1’s K422 and abrogating assembly of the CoREST complex (*204*). TRIM35 expression positively correlated with response to immune checkpoint blockade therapy (*204*). Importantly, TRIM35-depleted NSCLC cells could be sensitized to PD-1 blockade through LSD1 inhibitors, which further increase GZMB^+^CD8^+^ T cell tumor infiltration (*204*). Last, LSD1 knockdown or inhibition increases expression of chemokines that attract CD8^+^ T cells in highly aggressive triple-negative breast cancer (TNBC) cells (*205*). LSD1 inhibitors also enhanced antitumor activity of PD-1 blockade therapy in mice bearing TNBC tumors and PD-L1 blockade in a cervical cancer allograft model (*205*, *206*).

As previously mentioned, LSD1 phosphorylation at S111 is important for its nuclear localization and contributes to EMT and CSC phenotypes in breast cancer (*69*). LSD1-S111p is highly expressed in CD8^+^ T cells of patients with immunotherapy-resistant melanoma and resistant 4T1 TNBC mice (*207*). Compared to effector and memory CD8^+^ T cells, the transcription factor EOMES is more highly expressed in exhausted CD8^+^ T cells, which are associated with resistance to immunotherapy (*208*, *209*). About 63% of CD8^+^ T cells in patients with immunotherapy-resistant melanoma were LSD1^+^EOMES^+^ versus only 30% in responder patients (*207*). Interestingly, LSD1 demethylase activity maintains EOMES in a K373 hypomethylated/K641 hyperacetylated state in the nucleus, which contributes to the exhausted state (*207*). A total of 70% of CD8^+^ tumor–infiltrating T cells in resistant patients expressed the EOMES K641ac form versus only 5% in responders, whereas the methylated form was expressed in 54% of responders versus 23% of resistant patients (*207*). Importantly, EPI-111, an LSD1-S111p–specific inhibitor that abrogates LSD1-S111p nuclear localization, increased EOMES protein methylation, inhibited mesenchymal stem–like markers, and induced CD8^+^ T cell infiltration in 4T1 TNBC in vivo (*207*). Targeting the CoREST complex through bifunctional molecules bearing both LSD1 and HDAC inhibitor moieties abrogated regulatory T cell (T_reg_) suppressive function and promoted CD8^+^ T cell tumor infiltration in the TC1 lung cancer mouse model (*210*). In line with this, wild-type (WT) or TC1 mice engineered with a conditional *RCOR1* knockout in T_regs_ had reduced peripheral T_reg_ numbers and impaired organ allograft tolerance (WT mice) and had substantial reduction in lung tumor growth (TC1 mice) (Fig. 8C) (*210*). Mechanistically, the CoREST complex associates with Foxp3 and is necessary for suppression of IL-2 and IFN-γ production and maintenance of the T_reg_ phenotype (*210*). Conditional deletion of HDAC1 in T_regs_ impaired their suppressive function, whereas deletion of HDAC2 had the opposite effect (Fig. 8C) (*211*). Notably though, pharmacological targeting of HDAC1-3 with entinostat did not affect T_reg_ function (*212*). One explanation for this discrepancy is that eradication of noncatalytic properties of HDAC1 through protein depletion is necessary to abrogate T_reg_ suppressive function, although lack of a phenotype with entinostat may also be simply due to cotargeting of two HDACs that have opposing individual effects.

## CoREST COMPLEX INHIBITORS/DEGRADERS IN CLINICAL DEVELOPMENT

CoREST complex inhibitors or degraders are now being assessed in clinical trials for treatment of solid and hematologic cancers. Table 1 summarizes ongoing trials investigating LSD1/HDAC1 inhibitors or a CoREST complex degrader (UM171).

**Table 1.** CoREST complex–targeting molecules now in clinical development. Clinical trials investigating CoREST complex–targeting molecules are summarized, highlighting their indications, efficacy, trial phases, and the most common treatment-emergent adverse events. GVHD, graft-versus-host disease; CSF, colony-stimulating factor.

Trial number	Status	CoREST complex–targeting molecules	Combination treatment	Indications	Phase	Efficacy	Most common treatment-emergent adverse events
NCT04081220	Recruiting	Bomedemstat (IMG-7289)^*^		Essential thrombocythemia	2		
NCT05558696	Recruiting			Polycythemia vera	2		
NCT05597306	Recruiting		Venetoclax	AML	1		
NCT05569538	Recruiting			Myelofibrosis	2		
NCT02842827	Completed (*245*)		Tretinoin	AML, MDS	1|2	Overall response rate of 28.2% in AML	Diarrhea (42%), nausea (42%), and thrombocytopenia (38%)
NCT04262141	Recruiting			Essential thrombocythemia|polycythemia vera	2		
NCT04254978	Completed (*213*)			Essential thrombocythemia	2	95% had reduced platelet counts to ≤400 × 10^9^/liter in a median of 10 weeks	Dysgeusia (55%), constipation (38%), thrombocytopenia (34%), and arthralgia (27%)
NCT03136185	Completed (*246*, *247*)			Myelofibrosis	1|2	72% had a reduction in the total symptom score, 64% had a reduction in the spleen volume from the baseline	Dysgeusia (36%), diarrhea (34%), thrombocytopenia (29%), and anemia (22%)
NCT05191797	Recruiting		Atezolizumab	Extensive-stage SCLC|limited-stage SCLC	1|2		
NCT04748848	Terminated (no results posted)	CC-90011^*^	Venetoclax, azacitidine	AML	1		
NCT04350463	Completed (*216*)		Nivolumab	SCLC or squamous NSCLC	2	Overall response rate of 5.7–10.3%	Anemia (51.1%), thrombocytopenia (46.6%), decreased appetite (29.5%), and asthenia (26.1%)
NCT04628988	Completed (no results posted)			Prostatic neoplasms	1		
NCT02875223	Terminated (*215*)			Lymphoma, non-Hodgkin neoplasms	1	Overall response rate of 4%	Fatigue (48%) and thrombocytopenia (46%)
NCT02034123	Terminated (*219*)	GSK2879552^*^		SCLC	1	Poor disease control. Unfavorable risk-to-benefit ratio	Thrombocytopenia (41%). 14% of patients developed encephalopathy leading to one death and study termination.
NCT02177812	Terminated (*218*)		Tretinoin	AML	1	Unfavorable risk-to-benefit ratio	Febrile neutropenia (54%), nausea (46%), hypokalemia (41%), and thrombocytopenia (20%)
NCT05546580	Recruiting	Iadademstat (ORY-1001)^*^	Gilteritinib	AML	1		
NCT05420636	Recruiting		Paclitaxel	SCLC	2		
NCT06287775	Recruiting		Atezolizumab or durvalumab	Extensive-stage SCLC	1|2		
NCT03132324	Terminated	INCB059872^*^		Sickle cell disease	1	Terminated due to business decisions	
NCT03514407	Terminated			Relapsed Ewing sarcoma	1		
NCT02959437	Terminated		Pembrolizumab and epacadostat	Solid tumors|advanced malignancies|metastatic cancer	1|2		
NCT02712905	Terminated		Azacitadine and tretinoin	Solid tumors and hematologic malignancy	1|2		
NCT05268666	Recruiting	JBI-802^*^		Locally advanced solid tumor|metastatic solid tumor	1|2		
NCT03505528	Completed (*248*)	Phenelzine sulfate^*^	Paclitaxel	Metastatic breast cancer	1		Dizziness (7%), fatigue (5%), neutropenia (3%), and peripheral neuropathy (3%)
NCT04611139	Withdrawn	Seclidemstat (SP-2577)^*^	Pembrolizumab	Gynecologic cancers	1		
NCT05266196	Enrolling_by_invitation			Ewing or Ewing-related sarcomas	1|2		
NCT03600649	Active_not_recruiting		Topotecan and cyclophosphamide	Ewing or Ewing-related sarcomas	1		
NCT04734990	Recruiting		Azacitidine	MDS or chronic myelomonocytic leukemia	1|2		
NCT03895684	Completed (*249*)			Advanced solid tumors	1	54% had the best response of stable disease with a median time to progression of 4.3 months	Diarrhea (5.3%) and abdominal pain (5.3%)
NCT03228433	Terminated (*228*)	TAK-418^*^		Healthy volunteers	1		Headache (50%), nausea (22%), and decreased appetite (17%)
NCT03501069							
NCT02273102	Completed (*250*)	TCP^*^	Tretinoin	AML	1	Overall response rate of 23.5% (in combination with ATRA)	Fatigue (35%), creatinine increased (29%), dizziness (29%), dry mouth (29%), and headache (29%)
NCT02261779	Terminated (*229*)		Tretinoin	AML	1|2	Refractory/progressive disease and infectious complications led to termination	Vertigo (39%) and hypotension (22%)
NCT02717884	Unknown (*251*)		Tretinoin low-dose cytarabine	AML, MDS	1|2	Partial remission (8%)	Thrombocytopenia (45.8%) and neutropenia (20.8%)
NCT05887492	Recruiting	TNG260^†^	Pembrolizumab	*STK11*-mutated solid tumors	1|2		
NCT04594031	Withdrawn	UM171^‡^		Sickle cell disease|umbilical cord blood|hematopoietic cell proliferation	1		
NCT03913026	Active_not_recruiting			High-risk hematologic malignancy|cord blood transplant	2		
NCT03441958	Active_not_recruiting			Multiple myeloma	1|2		
NCT04990323	Recruiting			High-risk myeloid malignancies|cord blood transplant	1|2		
NCT04103879	Active_not_recruiting			High-risk hematological malignancy|cord blood transplant	2		
NCT02668315	Completed (*233*, *236*)			Hematologic malignancy	1|2	Probability of nonrelapse mortality: 4.5% (UM171) versus 30% (unmanipulated cord blood); probability of GVHD-free, relapse-free survival: 63.6% (UM171) versus 27.9% (unmanipulated cord blood)	Grade 3 febrile neutropenia (73%) and bacteremia (41%)
NCT04932291	Completed (*252*)	Vafidemstat (ORY-2001)^*^		Borderline personality disorder	2	58.6% reduction in the STAXI-2 Trait Anger scale (agitation and aggression)	Headache (12.3%), nasopharyngitis (8.5%), and reduced platelet counts (7.5%)
NCT03867253	Completed (*232*)			Mild to moderate Alzheimer’s disease	2	Reduction of proinflammatory YKL40 and NFL levels in CSF. Substantial reduction in agitation-aggression after 12-month treatment.	Safe and well tolerated. Specific safety data are not posted.

*LSD1 inhibitor.

†HDAC1 inhibitor.

‡CoREST complex degrader.

Bomedemstat, an irreversible LSD1 inhibitor, reduced platelet counts to ≤400 × 10^9^/liter in the absence of new thromboembolic events in 95% of patients (61 of 64) with essential thrombocythemia (*213*). In addition, 85% of patients (39 of 46) had a decrease in frequencies of mutant *JAK2*, *CALR*, and *MPL* alleles (*213*).

CC-90011, a potent and reversible oral LSD1 inhibitor, was well tolerated in 50 patients with solid tumors (49) and relapsed/refractory (R/R) non-Hodgkin lymphoma (1) with thrombocytopenia being the most common adverse events reported (40%) (*214*). Patients with NE tumors receiving CC-90011 had a stable disease for at least 6 months, and a complete response was observed in the patient with R/R non-Hodgkin lymphoma (*214*). A long-term safety and efficacy study of CC-90011 showed reduced expression of NE markers, although the overall response rate was 4% (*215*). CC-90011 safety and preliminary efficacy has also been assessed in combination with standard-of-care cisplatin and etoposide with or without nivolumab in patients with first-line extensive-stage SCLC with an overall response rate of 5.7 to 10.3% (*216*). The potential capability of CC-90011 to restore AR protein expression (measured through FDG/FDHT positron emission tomography imaging) and, consequently, sensitivity to antihormonal therapy in CRPC are also being tested (*217*). Clinical trials evaluating GSK2879552 in patients with R/R myelodysplastic syndrome and AML or SCLC were terminated because of the high risk-to-benefit ratio, with reported toxicities such as hemorrhage and grade 3/4 thrombocytopenia (*218*, *219*).

Iadademstat also had a good safety profile in patients with R/R AML, although thrombocytopenia was a frequently reported adverse event (*220*). Importantly, iadademstat induced morphologic and molecular differentiation in leukemic blasts, especially in patients with *MLL* gene rearrangements (four of five patients, 80%). Iadademstat + azacitidine treatment, assessed as a first-line treatment in elderly unfit patients with AML (phase 2a ALICE trial), resulted in an 81% objective response rate (64% complete remission and 36% partial remission) with a manageable toxicity profile (*221*). Iadademstat is also now being evaluated in combination with paclitaxel in patients with R/R SCLC, with atezolizumab in patients with extensive-stage SCLC and with gilteritinib in patients with R/R FLT3-mutant AML.

JBI-802 is a dual LSD1 and HDAC6/8 inhibitor that has shown a good safety profile and preliminary efficacy in two patients with immunotherapy-refractory NSCLC (*222*). Seclidemstat or SP-2577 is a potent and reversible LSD1 inhibitor that inhibits both LSD1’s catalytic and scaffolding properties. Seclidemstat is being tested as a monotherapy for treatment of patients with R/R Ewing sarcoma or advanced solid cancers (*223*, *224*), or in combination with azacitidine for patients with myelodysplastic syndrome or chronic myelomonocytic leukemia (*225*). Seclidemstat has shown a manageable safety profile in heavily pretreated patients with R/R Ewing sarcoma with a 60% objective response rate in patients with first relapse (*226*, *227*).

As mentioned earlier, TAK-418 showed a therapeutic benefit in preclinical mouse models of autism spectrum disorders and AD. In phase 1 clinical trials, TAK-418 rapidly reached *C*_max_ in the cerebrospinal fluid and was well tolerated with favorable pharmacokinetic and pharmacodynamic profiles (*228*). Notably, no thrombocytopenia was observed in patients treated with TAK-418. A phase 1/2 trial assessed the safety and efficacy of TCP + ATRA therapy as a salvage therapy in 15 patients with R/R AML (*229*). TCP + ATRA had an acceptable safety profile with a 20% objective response rate, although the trial was terminated because of refractory/progressive disease and infectious complications. Vertigo and hypotension were the most common treatment-emergent adverse events (*229*).

TNG260 is a potent and selective HDAC1 inhibitor (10-fold selectivity to HDAC3), with 500-fold selectivity to HDAC1 in the CoREST complex compared to other HDAC1-containing complexes (*230*). TNG260 has shown durable antitumor activity in combination with PD-1 blockade therapy in several *STK11*-mutant tumor mouse models and is now being examined in clinical trials (*32*).

Consistent with the aforementioned results in AD preclinical models, the vafidemstat phase 2b PORTICO study showed statistically significant improvement in the secondary endpoint Borderline Evaluation of Severity, which is an overall measure of borderline personality disorder disease severity (*231*). Moreover, the REIMAGINE-AD and ETHERAL studies have shown that vafidemstat causes reductions in agitation/aggression scales and in cerebrospinal fluid levels of the inflammatory biomarker YKL40 in patients with mild-to-moderate AD (*232*).

To date, the KBTBD4-activating molecule UM171 is the only known degrader of all CoREST1/3 complex proteins. UM171-expanded cord blood grafts (ECT-001) are now being examined in phase 1/2 clinical trials with phase 3 expected soon for treatment of high-risk leukemias and myelodysplasias. A phase 1/2 trial showed that the single UM171-expanded cord blood transplants are feasible and safe with smaller cord blood units not compromising engraftment (*233*). In addition, because UM171 enables selection of smaller cord blood units, this increases cord blood availability to ethnic and racial minorities (*234*). Retrospective comparison of T cell reconstitution in patients who received ECT-001 versus patients who received unmanipulated cord blood transplants revealed a higher T cell repertoire diversity and lower frequency of severe infections in patients with UM171 at 12 months posttransplantation (*235*). Furthermore, compared to patients who received unmanipulated cord blood or matched-unrelated donor transplants, ECT-001 recipients had lower nonrelapse mortality rates and higher progression-free survival/graft-versus-host disease–free, relapse-free survival (*236*). Results from phase 2 trials in high and very high-risk patients with acute leukemias/myelodysplastic syndromes who received ECT-001 revealed high probabilities of 2-year overall survival (67%) and progression-free survival (63%) including in patients with p53 mutant (*237*).

While targeting LSD1 or CoREST complex components—including through clinical-stage agents like bomedemstat, iadademstat, CC-90011, and GSK2879552—has shown clinical activity across myeloid malignancies and solid tumors, it comes with notable and class-consistent side effects as mentioned. The most recurrent treatment-emergent adverse events observed in LSD1/CoREST-targeting trials include thrombocytopenia, dysgeusia, anemia, gastrointestinal toxicity (diarrhea and constipation), and neutropenia. Thrombocytopenia, in particular, was prominent across multiple studies (up to 47% incidence in some), suggesting on-target effects on megakaryopoiesis due to disruption of the LSD1-GFI1/1B axis.

For instance, in the completed phase 2 trial of bomedemstat (NCT04254978), adverse events such as dysgeusia (55%), thrombocytopenia (34%), and arthralgia (27%) were frequent. Similarly, GSK2879552, as mentioned above, led to encephalopathy and thrombocytopenia in 41% of patients, with one fatality leading to trial termination. These hematologic and neurologic effects underscore the potential for toxicity when modulating epigenetic enzymes broadly expressed in normal hematopoietic and neural cells.

UM171 has only been tested ex vivo in clinical trials to expand cord blood stem cells for transplantation. The observed side effects in these trials—such as febrile neutropenia and bacteremia—are therefore related to the transplant context rather than UM171 itself. No signal of thrombocytopenia or neurotoxicity has emerged in this ex vivo setting.

In summary, while LSD1-targeting agents demonstrate promising efficacy, especially in hematologic diseases, the predictable on-target toxicities—particularly cytopenias and taste disturbances—are dose limiting. This highlights the need for a more tightly controlled approach when targeting CoREST complex proteins to circumvent some of these liabilities whether through substantial but incomplete protein degradation (as is the case with UM171) or through selective modulation of catalytic or scaffolding properties. Careful monitoring for hematologic and neurologic complications remains essential in future clinical translation of UM171 and other LSD1/CoREST complex inhibitors.

## MECHANISMS OF RESISTANCE TO LSD1 INHIBITORS

A limited number of studies have described mechanisms of resistance to LSD1 inhibitors, while it remains unknown whether resistance can develop to inhibition of HDAC1/2 within CoREST complexes. In SCLC, sensitivity to LSD1 inhibitors is linked to neuroendocrine transcriptional programs, whereas resistance correlates with a TEAD4-driven mesenchymal-like state as mentioned earlier (*113*). This highlights transcriptional plasticity and lineage switching as key mediators of resistance. In AML, genome-wide CRISPR knockout screens have identified several components of the mTORC1 signaling pathway as synthetic lethal with LSD1 inhibition (*238*). Consistently, intrinsic resistance to LSD1 inhibitors involves activation of mTORC1 through IRS1/ERK signaling following treatment, and mTOR inhibition restores sensitivity (*239*). Moreover, lysine-specific demethylase 1 inhibitor–induced differentiation in AML requires the transcription factors PU.1 and C/EBPα; loss of either confers resistance, underscoring the importance of transcription factor networks in mediating response (*240*). In Ewing sarcoma, acquired resistance to the reversible LSD1 inhibitor SP-2509 occurs without the emergence of LSD1 mutations and is instead associated with transcriptional reprogramming, down-regulation of CoREST1, up-regulation of drug efflux transporters, and increased sensitivity to HDAC inhibitors—suggesting a shift in epigenetic dependency (*241*). CRISPR screens also revealed that disruptions in mitochondrial electron transport chain complexes III and IV conferred resistance to SP-2509 in Ewing sarcoma (*242*). Mechanistically, deletion of complex III and IV genes resulted in a transcriptional state similar to that of LSD1 inhibitor–treated cells, which prevented further regulation by the inhibitor (*242*). Together, these findings underscore the complexity of LSD1 inhibitor resistance and sensitivity and support the rationale for combination therapies targeting complementary pathways such as mTOR, HDACs, and mitochondrial metabolism. Whether similar mechanisms could arise with UM171 treatment remains to be seen.

## FUTURE DIRECTIONS

**Fig. 9.**
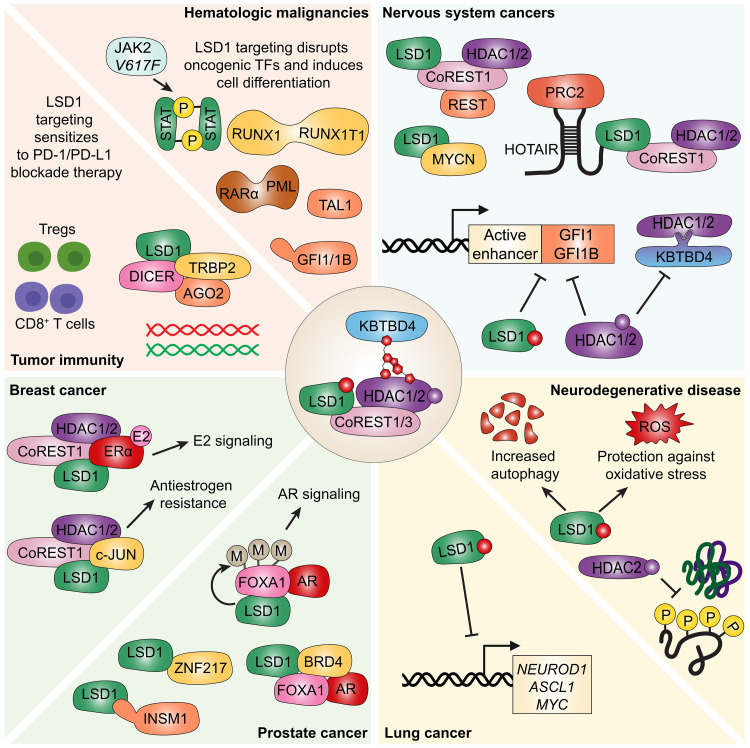
KBTBD4 and CoREST complex targeting: A new therapeutic frontier in oncology, neurodegeneration, and immunotherapy. The CoREST complex coopts different transcription factors or cofactors to elicit tumor- and tissue-specific transcriptional responses.

The CoREST complex functions as a versatile epigenetic regulator by partnering with context-specific transcription factors and cofactors—including REST, GFI1, ZNF217, INSM1, ERα, and AR—to orchestrate transcriptional programs that are essential for tumor progression, neuronal identity, and immune tolerance. Emerging evidence suggests that KBTBD4, an adaptor protein for the CRL3 E3 ubiquitin ligase complex, can redirect protein degradation machinery to selectively dismantle CoREST complex components. Recurrent neomorphic KBTBD4 mutations in MB phenocopy the activity of the small-molecule UM171, leading to CoREST degradation and transcriptional reprogramming. This positions KBTBD4 as a master regulator of CoREST complex stability and function, with broad influence over downstream epigenetic programs and cellular phenotypes. Figure 9 highlights the expanding therapeutic potential of targeting the CoREST complex across diverse disease contexts.

There is increasing evidence of the importance of both catalytic and scaffolding properties of LSD1 in therapeutic benefit seen with LSD1 inhibitors. The scaffolding properties of LSD1 appear to be crucial for GFI1-mediated differentiation blocks in AML. Reprogrammed breast cancer cells that have acquired resistance to long-term estrogen deprivation are sensitive to LSD1 knockout but not chemical inhibition. Similar data have also been reported in NE-CRPC cells. In prostate cancer, LSD1 inhibitors elicited antitumor responses either at high concentrations in short-term assays or after extended exposure (2 weeks) at physiological doses (*97*, *243*). This raises the interesting possibility that concomitant targeting of all CoREST complex proteins in prostate cancer may elicit faster and more durable responses compared to targeting LSD1 alone. Analysis of CRISPR data from DepMap reveals weak overall dependencies of HDAC1 and HDAC2 across cancer cell lines, likely because one compensates in the absence of the other. Cell lines of myeloid lineage are selectively dependent on CoREST1 for survival consistent with existing preclinical and clinical data on LSD1 inhibitors in AML, whereas LSD1 dependency does not appear to be selective across cancer lineages. This suggests that antitumor effects seen with LSD1 inhibitors in AML may be in part due to disruption of CoREST1 and/or associated transcription factors. Although this requires further investigation, it is plausible to hypothesize that simultaneous targeting of all CoREST complex proteins may elicit a stronger therapeutic response, particularly in cell lines that do not show selective dependency on individual targeting.

Recent developments support LSD1 targeting as a promising next-generation therapeutic approach in MPNs, with the potential to not only improve clinical outcomes but also shift the treatment paradigm toward eradication of the malignant clone and prevention of leukemic transformation. In MPNs, where epigenetic dysregulation drives clonal persistence and lineage skewing, activation of KBTBD4 may serve as a molecular rheostat—modulating stem and progenitor cell identity through degradation of the CoREST complex. Importantly, the outcome of CoREST complex depletion is context dependent: In normal HSCs, CoREST degradation enhances self-renewal and stem cell expansion, while in malignant or cancer cells, it promotes differentiation and cell death. Similar to LSD1 inhibition, KBTBD4 activation and consequent CoREST complex degradation may reprogram the transcriptional landscape that sustains mutant JAK2-, NFE2-, or CALR-driven stem cells, promoting differentiation and impairing disease propagation. Given that KBTBD4 activation enhances normal HSC fitness under stress, this approach is particularly relevant in MPNs, where malignant clones often outcompete normal hematopoiesis in an inflammatory, cytokine-rich marrow microenvironment (*244*). Thus, KBTBD4-targeting strategies could restore hematopoietic balance and potentially synergize with JAK inhibitors to eliminate malignant clones.

As targeted protein degradation continues to emerge as a powerful therapeutic modality, approaches targeting KBTBD4 (UM171) offer a promising and innovative way to selectively dismantle pathogenic epigenetic complexes. Together with pharmacologic LSD1 inhibition, these strategies represent complementary avenues for disarming the CoREST complex in diseases driven by epigenetic dysfunction—including cancer, neurodegeneration, and immune exhaustion.
